# Supplementary material for: The PqsE Active Site as a Target for Small Molecule Antimicrobial Agents against Pseudomonas aeruginosa
Source: Biochemistry. 2022 Aug 19;61(17):1894–903. doi: 10.1021/acs.biochem.2c00334 (PMC9454246; doi:10.1021/acs.biochem.2c00334)
Supplement: Supplementary file 1 — bi2c00334_si_001.pdf [file bi2c00334_si_001.pdf]

Supporting Information for

The PqsE active site as a target for small molecule antimicrobial agents against *Pseudomonas aeruginosa*

Isabelle R. Taylor[1], Philip D. Jeffrey[1], Dina A. Moustafa[2], Joanna B. Goldberg[2], Bonnie L. Bassler[1,3*]

[1]Department of Molecular Biology, Princeton University, Princeton, NJ 08544, USA.
[2]Emory University School of Medicine, Children's Healthcare of Atlanta, Inc., Department of Pediatrics, and Center for Cystic Fibrosis and Airway Diseases Research, Atlanta, GA 30322, USA.
[3]Howard Hughes Medical Institute, Chevy Chase, MD 20815, USA.

Bonnie Bassler
Email:bbassler@princeton.edu

This PDF file includes:

Figures S1 to S7
Tables S1 and S2
Supplementary text

Supplementary Figures

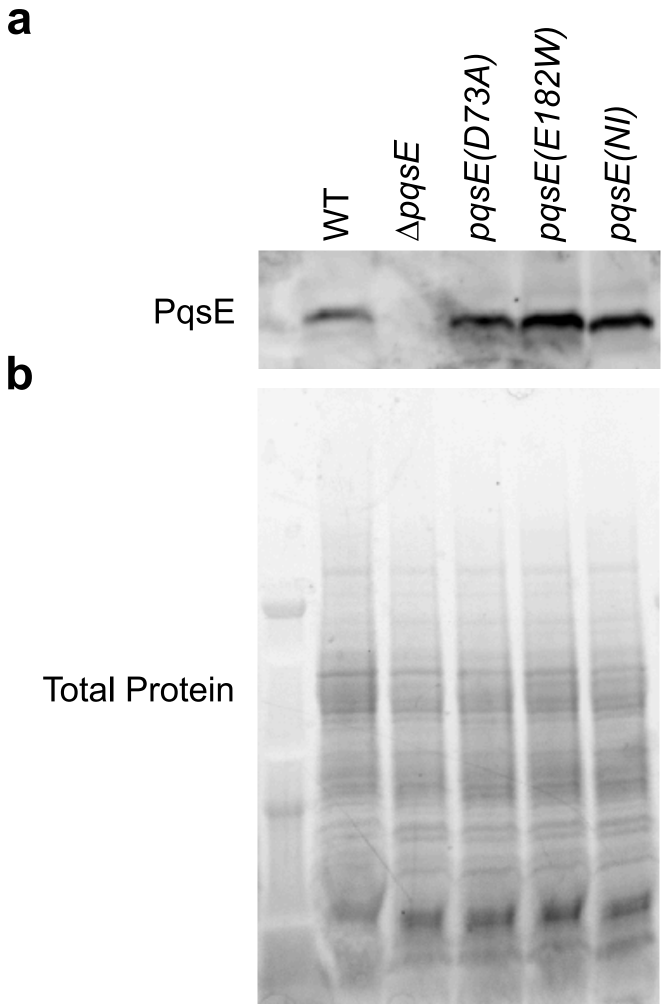

Fig. S1. Western blot analysis of PqsE proteins made by *P. aeruginosa* PA14. *P. aeruginosa* PA14 harboring the designated *pqsE* alleles at the native *pqsE* locus were grown to OD_{600} = 1.5, lysed, and subjected to SDS-PAGE and Western blot analysis. a) Western blot of the gel in panel b following incubation with a primary rabbit polyclonal α-PqsE antibody and secondary α-rabbit HRP-conjugated antibody. Detection was via chemiluminescence. b) Total protein was imaged using a UV-activated in-gel protein stain.

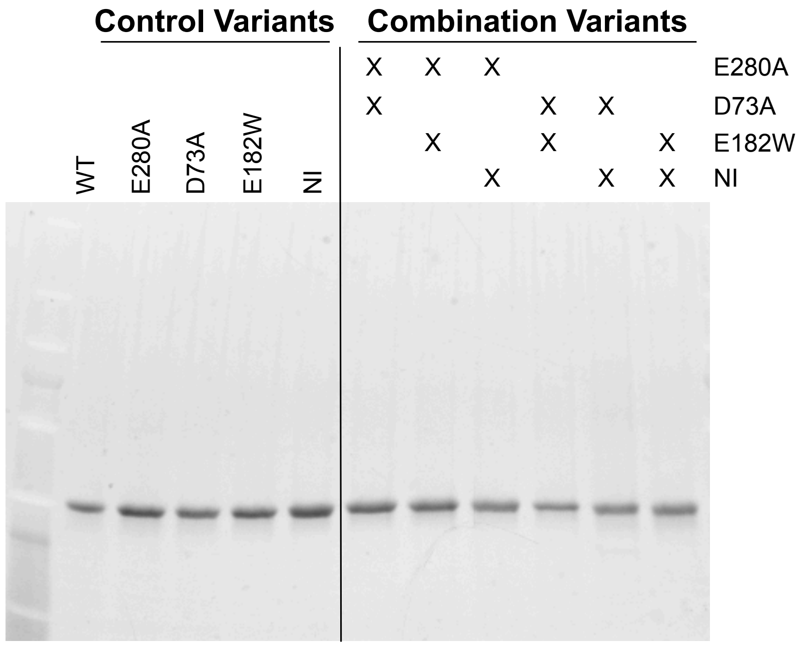

Fig. S2. SDS-PAGE analysis of purified PqsE proteins. Approximately 0.6 μg of the specified purified PqsE protein was loaded into each lane and imaged using a UV-activated in-gel protein stain. For the combination variants, “X” signifies that the protein in that lane harbors the corresponding substitution indicated on the right. All purified PqsE proteins appeared as ~34 kDa bands on the gel.

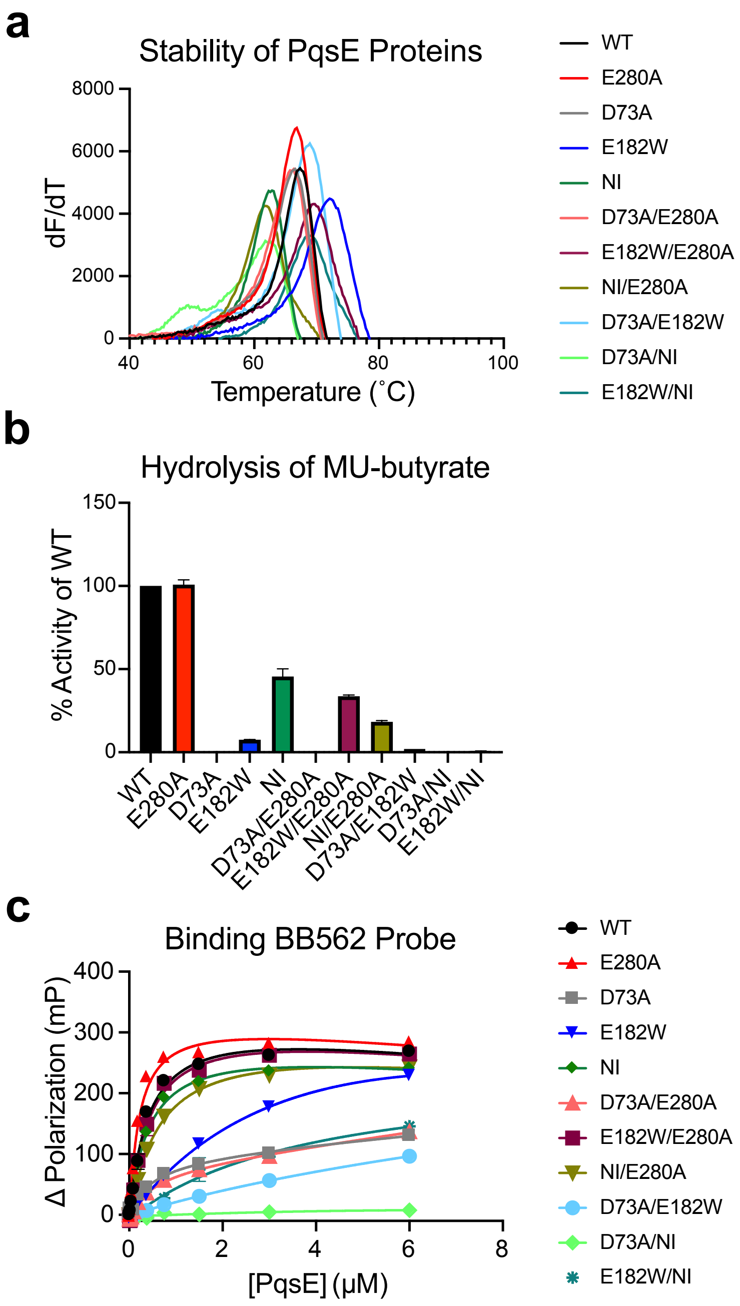

Fig. S3. Biochemical characterization of purified PqsE variants. a) First derivative plots of melt curves for PqsE proteins. All proteins were tested at 5 μM with 200 μM $MnCl_2$. dF/dT indicates the change in SYPRO Orange fluorescence divided by the change in temperature. The peak of each curve is interpreted as the T_m. Each melt curve was performed in triplicate and the first derivative was taken for the resulting averaged melt curves. b) Rate of hydrolysis of the substrate MU-butyrate (2 μM) was determined for each purified PqsE protein at 100 nM over 2.5 min. Results are the average of two independent experiments performed in triplicate. Rates are normalized to that of the WT PqsE protein (100%). c) Binding of PqsE proteins to the BB562 active site fluorescent probe in the presence of 2 μM $MnCl_2$ was measured as the change in fluorescence polarization. Results are the average of two independent experiments performed in triplicate. Error bars represent standard deviations.

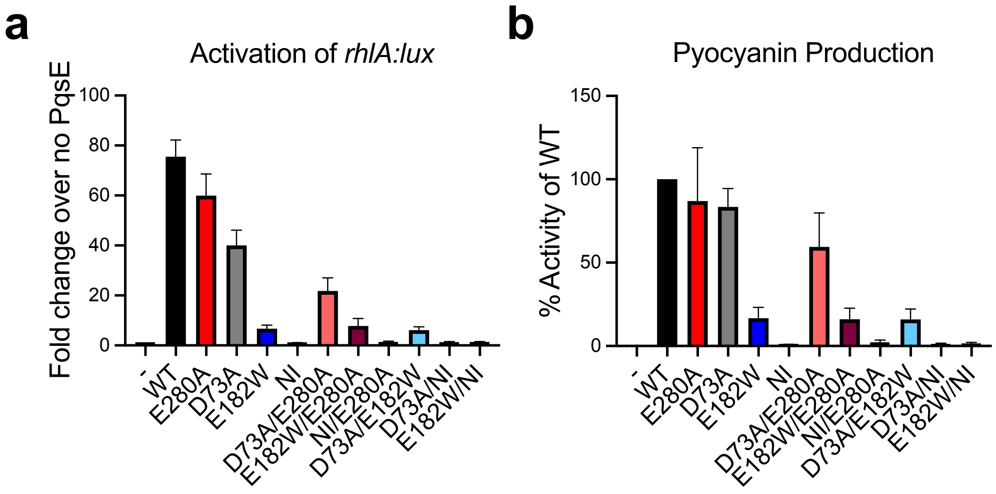

Fig. S4. *In vivo* characterization of PqsE proteins. a) Activation of RhlR transcription factor activity by production of RhlR and PqsE proteins in *E. coli*. All strains were grown in the presence of 100 nM C4-HSL. The bioluminescence measurements were divided by the OD_{600} measurements and are represented as the fold increase compared to the no PqsE strain ("-"). Results are the average of 2 biological replicates performed in technical triplicate. b) Pyocyanin production was measured for strains expressing the *pqsE* allele from the pUCP18 plasmid. The OD_{695} values of the cell-free culture fluids were measured and divided by the OD_{600} values of the cultures. The results were normalized to that of the strain producing WT PqsE (100%). Results are the average of 2 biological replicates. Error bars represent standard deviations.

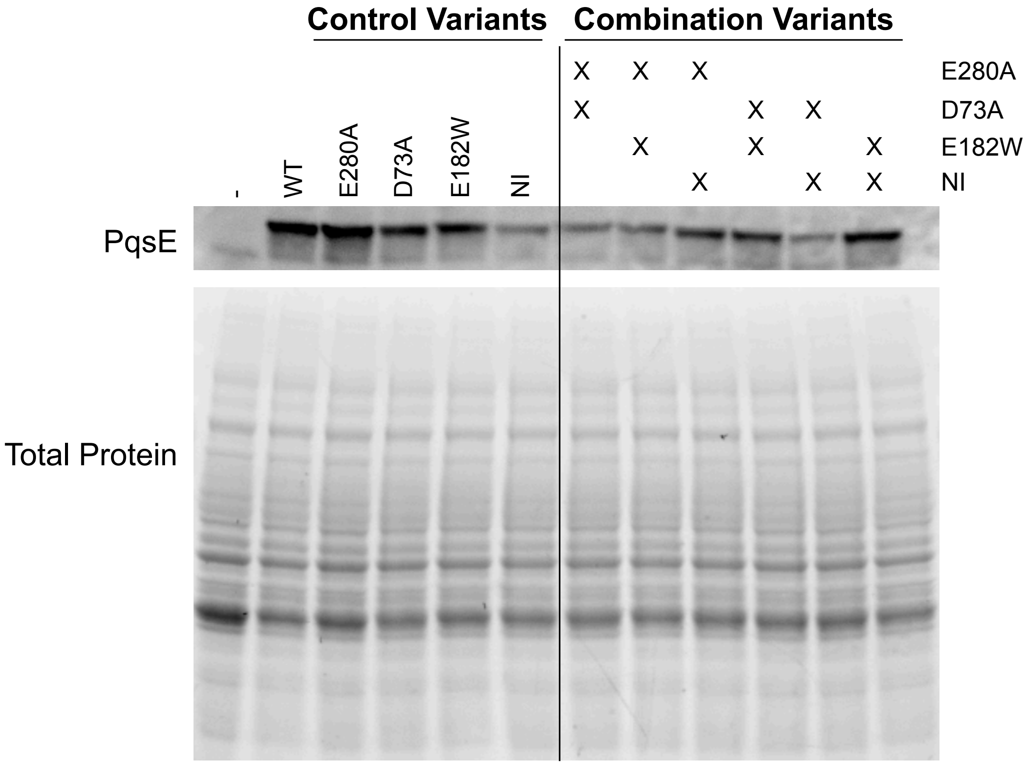

Fig. S5. Western blot analysis of PqsE proteins present in *E. coli* lysates. Lysates from *E. coli* strains producing the indicated PqsE protein from the pACYC184 plasmid (or harboring the empty vector, designated "-") were prepared. Top: Western blot of the gel (shown on the bottom) following incubation with a primary rabbit polyclonal α-PqsE antibody and secondary α-rabbit HRP-conjugated antibody. Detection was via chemiluminescence. Bottom: Total protein was imaged using a UV-activated in-gel protein stain. For the combination variants, "X" signifies that the variant in that lane harbors the corresponding substitution indicated on the right.

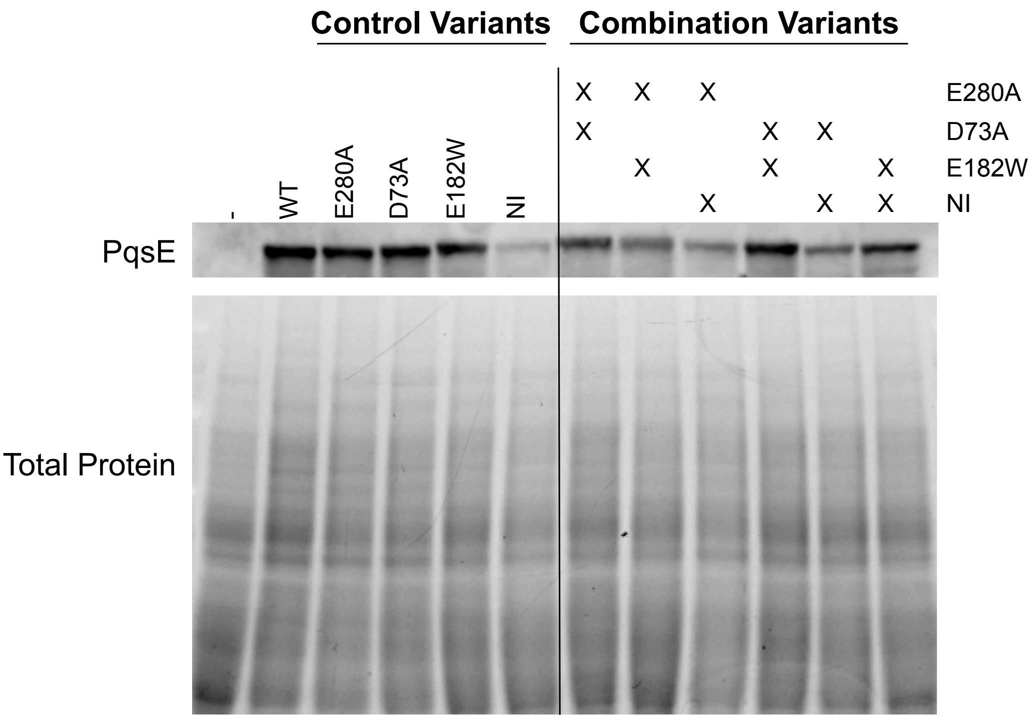

Fig. S6. Western blot analysis of *P. aeruginosa* PA14 lysate. Lysates were prepared from *P. aeruginosa* PA14 strains harboring *pqsE* variants on the pUCP18 plasmid (or the empty vector, designated "-"). Top: Western blot of the gel (shown in the bottom) following incubation with a primary rabbit polyclonal α-PqsE antibody and secondary α-rabbit HRP-conjugated antibody. Detection was via chemiluminescence. Bottom: Total protein was imaged using a UV-activated in-gel protein stain. For the combination variants, "X" signifies that the variant in that lane harbors the corresponding substitution indicated on the right.

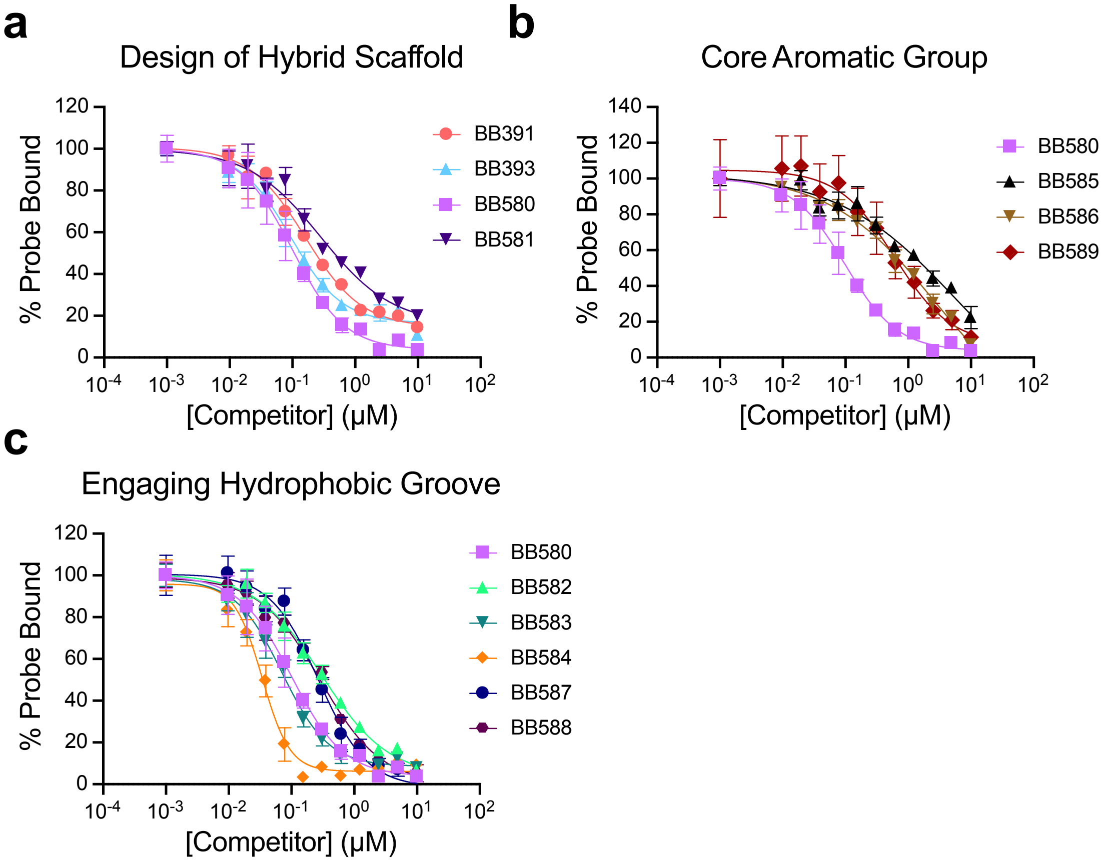

Fig. S7. Structure-activity relationships for BB391-BB393 hybrid derivatives. a) Optimization of "position 1", the amide bond orientation. b) Optimization of "position 2", the core aromatic group. c) Optimization of "position 3", the hydrophobic tail. Competition curves with the BB562 active site fluorescent probe were fit from the average of triplicates and error bars represent standard deviations. Polarization value for PqsE-BB562 in the absence of competitor is defined as 100% Probe Bound. All polarization values were background-subtracted by the reading for the probe in the absence of PqsE (background fluorescence).

Supplementary Tables

Strain	Description	Reference
UCBPP-PA14	PA14 *P. aeruginosa* Wildtype	Laboratory stock
SM52	PA14 Δ*rhlI*	24
SM101	PA14 Δ*rhlI* Δ*rhlR*	This study
SM381	PA14 *PrhlA-mNeonGreen*	24
SM563	PA14 Δ*pqsE PrhlA-mNeonGreen*	25
SM776	*E. coli* BL21 (DE3) pET28b-6xHis-pqsE(WT)	25
SM1328	*E. coli* Top10 P_{BAD}-*rhlR PrhlA-luxCDABE pACYC184(control)*	20
JPS0103	*E. coli* BL21 (DE3) pET23-rhlR	27
JPS0237	*E. coli* BL21 (DE3) pET28b-6xHis-pqsE(NI)	21
IT42	PA14 Δ*pqsE PrhlA-mNeonGreen pUCP18-*P_{lac}*-pqsE(WT)*	20
IT53	*E. coli* BL21 (DE3) pET28b-6xHis-pqsE(E182W)	20
IT68	PA14 Δ*pqsE PrhlA-mNeonGreen pUCP18(control)*	20
IT69	PA14 Δ*pqsE PrhlA-mNeonGreen pUCP18-*P_{lac}*-pqsE(E182W)*	20
IT88	*E. coli* BL21 (DE3) pET28b-6xHis-pqsE(D73A)	20
IT89	PA14 Δ*pqsE PrhlA-mNeonGreen pUCP18-*P_{lac}*-pqsE(D73A)*	20
IT100	PA14 *pqsE(E182W)*	This study
IT105	PA14 Δ*pqsE*	This study
IT106	PA14 *pqsE(D73A)*	This study
IT107	*E. coli* BL21 (DE3) pET28b-6xHis-pqsE(D73A/E182W)	This study
IT110	*E. coli* BL21 (DE3) pET28b-6xHis-pqsE(E182W/E280A)	This study
IT111	PA14 *pqsE(NI)*	This study
IT114	*E. coli* BL21 (DE3) pET28b-6xHis-pqsE(E280A)	This study
IT116	*E. coli* Top10 P_{BAD}-*rhlR PrhlA-luxCDABE pACYC184_DP-*P_{lac}*-pqsE(WT)*	21
IT117	*E. coli* Top10 P_{BAD}-*rhlR PrhlA-luxCDABE pACYC184_DP-*P_{lac}*-pqsE(D73A)*	21
IT118	*E. coli* Top10 P_{BAD}-*rhlR PrhlA-luxCDABE pACYC184_DP-*P_{lac}*-pqsE(E182W)*	21
IT119	*E. coli* Top10 P_{BAD}-*rhlR PrhlA-luxCDABE pACYC184_DP-*P_{lac}*-pqsE(NI)*	21
IT120	*E. coli* Top10 P_{BAD}-*rhlR PrhlA-luxCDABE pACYC184_DP-*P_{lac}*-pqsE(D73A/E182W)*	This study
IT121	*E. coli* Top10 P_{BAD}-*rhlR PrhlA-luxCDABE pACYC184_DP-*P_{lac}*-pqsE(E182W/E280A)*	This study
IT122	PA14 Δ*rhlI* ΔpqsE	This study
IT123	PA14 Δ*rhlI pqsE(D73A)*	This study
IT124	PA14 Δ*rhlI pqsE(E182W)*	This study
IT129	PA14 Δ*rhlI pqsE(NI)*	This study
IT130	PA14 Δ*pqsE PrhlA-mNeonGreen pUCP18-*P_{lac}*-pqsE(E280A)*	This study
IT131	PA14 Δ*pqsE PrhlA-mNeonGreen pUCP18-*P_{lac}*-pqsE(D73A/E182W)*	This study
IT132	PA14 Δ*pqsE PrhlA-mNeonGreen pUCP18-*P_{lac}*-pqsE(E182W/E280A)*	This study
IT133	PA14 Δ*pqsE PrhlA-mNeonGreen pUCP18-*P_{lac}*-pqsE(NI)*	This study
IT139	*E. coli* BL21 (DE3) pET28b-6xHis-pqsE(D73A/E280A)	This study
IT140	*E. coli* BL21 (DE3) pET28b-6xHis-pqsE(NI/E280A)	This study
IT141	PA14 Δ*pqsE PrhlA-mNeonGreen pUCP18-*P_{lac}*-pqsE(D73A/E280A)*	This study
IT142	PA14 Δ*pqsE PrhlA-mNeonGreen pUCP18-*P_{lac}*-pqsE(NI/E280A)*	This study
IT143	*E. coli* Top10 P_{BAD}-*rhlR PrhlA-luxCDABE pACYC184_DP-*P_{lac}*-pqsE(E280A)*	This study
IT160	*E. coli* BL21 (DE3) pET28b-6xHis-pqsE(D73A/NI)	This study
IT161	*E. coli* BL21 (DE3) pET28b-6xHis-pqsE(E182W/NI)	This study
IT164	PA14 Δ*pqsE PrhlA-mNeonGreen pUCP18-*P_{lac}*-pqsE(D73A/NI)*	This study
IT165	PA14 Δ*pqsE PrhlA-mNeonGreen pUCP18-*P_{lac}*-pqsE(E182W/NI)*	This study
IT167	*E. coli* Top10 P_{BAD}-*rhlR PrhlA-luxCDABE pACYC184_DP-*P_{lac}*-pqsE(D73A/NI)*	This study
IT168	*E. coli* Top10 P_{BAD}-*rhlR PrhlA-luxCDABE pACYC184_DP-*P_{lac}*-pqsE(E182W/NI)*	This study

Table S1. Strains used in this study.

	E182W	E182W-E280A	BB584
**PDB accession**	7TZ9	7U6G	7TZA
**Data collection**			
**Space group**	*P3₂21*	*P3₂21*	*P3₂21*
**Cell dimensions**			
**a, b, c (Å)**	60.7, 60.7, 146.10	60.6, 60.6, 146.0	61.1, 61.1, 146.3
**α, β, γ (°)**	90., 90., 120.	90., 90., 120	90., 90., 120
**Resolution**	29 – 2.01	30 – 2.44	13 – 2.10
**Outer shell**	2.06 – 2.01	2.51 – 2.44	2.16 – 2.10
**Observations**	288714	116855	95747
**Unique reflections**	21945	12028	18595
**Completeness (%)**	99.8 (97.2)	99.2 (89.9)	97.0 (85.3)
**Multiplicity**	13.2 (13.2)	9.7 (7.8)	5.1 (2.8)
**R_{merge}**	0.079 (1.056)	0.094 (0.936)	0.075 (0.432)
**R_{meas}**	0.083 (1.100)	0.100 (1.000)	0.083 (0.518)
**<I/σ(I)>**	19.0 (2.7)	14.7 (1.8)	14.7 (2.6)
**$CC_{1/2}$**	0.998 (0.972)	0.999 (0.659)	0.998 (0.769)
**Refinement**			
**Resolution (Å)**	28 – 2.01	30 – 2.44	13 – 2.10
**R_{work}/R_{free}**	0.169/0.216	0.173/0.241	0.186/0.220
**No. atoms**			
**Protein**	2412	2332	2390
**Ligand/ion**	2	2	33
**Water**	109	34	103
**Average B-factor (Å^2)**	44.8	80.5	28.0
**RMSD**			
**Bond lengths (Å)**	0.006	0.007	0.007
**Bond angles (°)**	0.787	0.911	0.915
**Ramachandran**			
**Favored (%)**	96.9	96.5	97.3
**Outlier (%)**	0.0	0.0	0.0

Table S2. Crystallographic data collection and refinement statistics. Values in parentheses correspond to those for the outer shell resolution.

Supplementary Information Text
Synthetic Methods

Abbreviations

As specified below, the symbols and conventions used in these processes, schemes, and examples are consistent with those used in the contemporary scientific literature, for example, the Journal of the American Chemical Society or the Journal of Biological Chemistry. Specifically, the following abbreviations may be used in the examples:

ACN (acetonitrile)
Boc (*tert*-butoxycarbonyl)
CD_3OD (deuterated methanol)
$CDCl_3$ (deuterated chloroform)
DCM (dichloromethane)
DIEA (*N,N*-diisopropylethylamine)
DIPEA (*N,N*-diisopropylethylamine)
DMA (*N,N*-dimethylacetamide)
DMAP (4-dimethylaminopyridine)
DMF (*N,N*-dimethylformamide)
DMSO (dimethylsulfoxide)
DMSO-d_6 (deuterated dimethylsulfoxide)
Et_3N (triethylamine)
EtOAc (ethyl acetate)
EtOH (ethanol)
FA (formic acid)
g (grams)
h (hours)
H_2 (hydrogen gas)
HCl (hydrochloric acid)
HOBt (hydroxybenzotriazole)
L (liters)
$LiAlH_4$ (lithium aluminum hydride)
LiOH (lithium hydroxide)
M (molar)
MeOH (methanol)
mg (milligrams)
$MgSO_4$ (magnesium sulfate)
MHz (megahertz)
min (minutes)
mL (milliliters)
mm (millimeter)
mM (millimolar)
mmol (millimoles)
mol (moles)
MTBE (methyl *tert*-butyl ether)
N_2 (nitrogen gas)
Na_2SO_4 (sodium sulfate)
Pd/C (palladium on carbon)
$Pd(OH)_2$ (palladium hydroxide)
PE (petroleum ether)
psi (pounds per square inch)
RT (room temperature)
t-Bu (*tert*-butyl)
TEA (triethylamine)
TFA (trifluoroacetic acid)
THF (tetrahydrofuran)
μL (microliters)
μm (micron)
μmol (micromoles)
BOP [(Benzotriazol-1-yloxy)tris(dimethylamino)phosphonium hexafluorophosphate]
EDCI (*N*-(3-Dimethylaminopropyl)-*N'*-ethylcarbodiimide hydrochloride)
HATU (1-[bis(dimethylamino)methylene]-1*H*-1,2,3-triazolo[4,5-b]pyridinium 3-oxide hexafluorophosphate)

General Information

Unless otherwise indicated, all temperatures are expressed in °C (degrees Celsius). All reactions were conducted at room temperature (RT) unless otherwise noted. ^{1}H NMR spectra were recorded on a Varian VXR-400 or a Varian Unity-400 at 400 MHz field strength. Chemical shifts are expressed in parts per million (ppm, d units). Coupling constants (*J*) are in units of Hertz (Hz). Splitting patterns describe apparent multiplicities and are designated as s (singlet), d (doublet), t (triplet), q (quartet), m (multiplet), quin (quintet), or br (broad). Mass spectral analyses were performed on a Sciex API 100 instrument using electrospray ionization (ESI). The column used for chromatography was a Luna-C18 2.0*30mm, (3 μm particles). Detection methods are diode array (DAD). MS mode was positive electrospray ionization. MS range was 100-1000. Mobile phase A was 0.037% trifluoroacetic acid in water, and mobile phase B was 0.018% trifluoroacetic acid in HPLC grade acetonitrile (10-90%) over 2 min. Analytical thin layer chromatography was used to verify the purity as well as to follow the progress of reaction(s). Unless otherwise indicated, all final products were at least 95% pure as judged by HPLC/MS.

***N*-(4-(3-(2-methyl-2-morpholinobutyl)ureido)phenyl)-1*H*-indazole-7-carboxamide (BB580)**

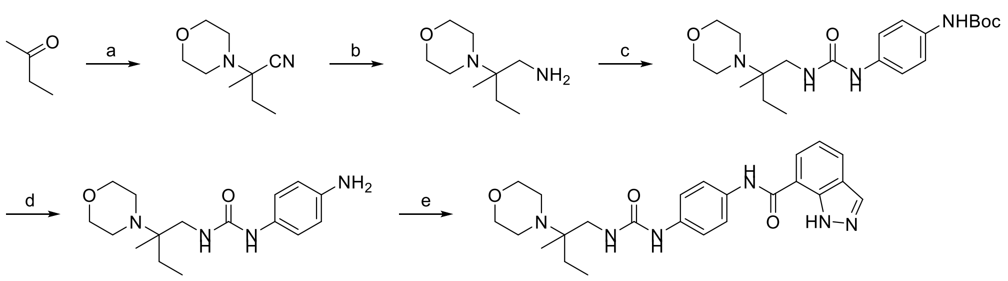

BB0580

Reagents and conditions: (a) morpholine, 2-hydroxy-2-methylpropanenitrile, $MgSO_4$, DMA, 45 °C, 12 h, 28% yield; (b) $LiAlH_4$, THF, 0 °C, 12 h, 54% yield; (c) triphosgene, *tert*-butyl (4-aminophenyl)carbamate, TEA, DCM, 0 °C, 2 h, crude; (d) HCl/MeOH, RT, 1 h, 6% yield for 2 steps; (e) 1*H*-indazole-7-carboxylic acid, DIEA, HATU, DMF, RT, 1 h, 30% yield for 2 steps.

Step a: 2-methyl-2-morpholinobutanenitrile

To a solution of morpholine (9.1 g, 104 mmol, 1.5 eq), butan-2-one (5.0 g, 69.34 mmol, 1.0 eq), and 2-hydroxy-2-methylpropanenitrile (5.9 g, 69.34 mmol, 1.0 eq) in DMA (10 mL) at RT under N_2(g) was added $MgSO_4$ (16.7 g, 138.69 mmol, 2.0 eq). The resulting reaction mixture was stirred at 45 °C for 12 h. The reaction mixture was poured into H_2O (50 mL) and extracted with EtOAc (3 x 50 mL). The combined organic phase was dried over anhydrous Na_2SO_4 and filtered, and the filtrate was concentrated under reduced pressure. The residue was purified by silica gel column chromatography (PE:EtOAc = 10:1 to 3:1) to afford 2-methyl-2-morpholinobutanenitrile (3.3 g, 28% yield) as a white solid. ^{1}H NMR (400 MHz, $CDCl_3$) δ 3.72 (t, *J* = 4.8 Hz, 4H), 2.64 (t, *J* = 4.8 Hz, 4H), 1.84 (q, *J* = 7.6 Hz, 2H), 1.45 (s, 3H), 1.02 (t, *J* = 7.6 Hz, 3H); LCMS calculated for $C_9H_{16}N_2O$: m/z = 168; found: m/z = 169 $(M+H)^+$.

Step b: 2-methyl-2-morpholinobutan-1-amine

To a solution of 2-methyl-2-morpholinobutanenitrile (1.5 g, 8.92 mmol, 1.0 eq) in THF (20 mL) at 0 °C under N_2(g) was added $LiAlH_4$ (1.18 g, 31.12 mmol, 3.49 eq). The resulting reaction mixture was stirred at RT for 12 h. The reaction mixture was poured into H_2O (50 mL) at 0 °C and extracted with EtOAc (3 x 50 mL). The combined organic phase was dried over anhydrous Na_2SO_4 and filtered, and the filtrate was concentrated under reduced pressure to afford 2-methyl-2-morpholinobutan-1-amine (820 mg, 54% yield) as a white solid. ^{1}H NMR (400 MHz, $CDCl_3$) δ 3.63 (m, 4H), 2.40 (m, 6H), 1.51 (m, 1H), 1.22 (m, 1H), 0.92 (d, *J* = 6.4 Hz, 3H), 0.83 (t, *J* = 7.6 Hz, 3H); LCMS calculated for $C_9H_{20}N_2O$: m/z = 172; found: m/z = 173 $(M+H)^+$.

Step c: *tert*-butyl (4-(3-(2-methyl-2-morpholinobutyl)ureido)phenyl)carbamate

To a solution of triphosgene (364 mg, 1.23 mmol, 0.33 eq) in DCM (5 mL) at 0 °C under N_2(g) was added a solution of *tert*-butyl (4-aminophenyl)carbamate (774 mg, 3.72 mmol, 1.0 eq) in DCM (5 mL). The resulting reaction mixture was stirred at 0 °C for 1 h. Then 2-methyl-2-morpholinobutan-1-amine (640 mg, 3.72 mmol, 1.0 eq) and TEA (752 mg, 7.43 mmol, 2.0 eq) were added, and the mixture was stirred at 0 °C for 1 h. The reaction mixture was poured into H_2O (50 mL) and extracted with EtOAc (3 x 30 mL). The combined organic phase was dried over anhydrous Na_2SO_4 and filtered, and the filtrate was concentrated under reduced pressure. The crude product was purified by reversed-phase HPLC (120 g Agela C18, 50 mL/min, water, 45-75% 20 min; 75% 10 min, Biotage) to afford *tert*-butyl (4-(3-(2-methyl-2-morpholinobutyl)ureido)phenyl)carbamate (2 g, crude) as a yellow solid. LCMS calculated for $C_{21}H_{34}N_4O_4$: m/z = 406; found: m/z = 407 $(M+H)^+$.

Step d: 1-(4-aminophenyl)-3-(2-methyl-2-morpholinobutyl)urea

To a solution of HCl/MeOH (4 M, 100 mL, 95.65 eq) at RT under N_2(g) was added *tert*-butyl (4-(3-(2-methyl-

2-morpholinobutyl)ureido)phenyl)carbamate (1.7 g, 4.18 mmol, 1.0 eq). The resulting reaction mixture was stirred at RT for 1 h. The reaction mixture was filtered, and the filtrate was concentrated under reduced pressure. The crude product was purified by reverse-phase preparative HPLC (column: Phenomenex Luna C18 200*40mm*10µm; mobile phase: [water(0.2%FA)-ACN]; B%: 1%-10%, 8 min) to afford 1-(4-aminophenyl)-3-(2-methyl-2-morpholinobutyl)urea (80 mg, 6% yield) as a black solid. ^{1}H NMR (400 MHz, CD_3OD) δ 8.26 (br, s, 1H), 7.10 (d, *J* = 8.4 Hz, 2H), 6.74 (d, *J* = 8.8 Hz, 2H), 3.81 (s, 4H), 3.01 (s, 4H), 1.68 (m, 2H), 1.18 (s, 3H), 0.99 (t, *J* = 7.6 Hz, 3H); LCMS calculated for $C_{16}H_{26}N_4O_2$: m/z = 306; found: m/z = 307 $(M+H)^+$.

Step e: *N*-(4-(3-(2-methyl-2-morpholinobutyl)ureido)phenyl)-1*H*-indazole-7-carboxamide (BB580)

To a solution of 1-(4-aminophenyl)-3-(2-methyl-2-morpholinobutyl)urea (50 mg, 163 µmol, 1.0 eq) and 1*H*-indazole-7-carboxylic acid (40 mg, 244 µmol, 1.5 eq) in DMF (1 mL) at RT under N_2(g) was added DIEA (53 mg, 407 µmol, 2.5 eq) and HATU (93 mg, 244 µmol, 1.5 eq). The resulting reaction mixture was stirred at RT for 1 h. The reaction mixture was filtered, and the filtrate was purified by reverse-phase preparative HPLC (column: Waters Xbridge BEH C18 100*30mm*10µm; mobile phase: [water(10mM NH_4HCO_3)-ACN]; B%: 20%-50%,10 min) to afford *N*-(4-(3-(2-methyl-2-morpholinobutyl)ureido)phenyl)-1*H*-indazole-7-carboxamide (33.4 mg, 30% yield, 100% purity by LCMS) as a white solid. ^{1}H NMR (400 MHz, DMSO-d_6) δ 13.11 (br, s, 1H), 10.23 (br, s, 1H), 8.63 (s, 1H), 8.12 (s, 1H), 7.96 (m, 2H), 7.59 (d, *J* = 8.8 Hz, 2H), 7.33 (d, *J* = 8.8 Hz, 2H), 7.18 (t, *J* = 7.6 Hz, 1H), 5.86 (t, *J* = 5.2 Hz, 1H), 3.53 (t, *J* = 4.4 Hz, 4H), 3.25 (s, 6H), 3.04 (dd, *J* = 18.4, 5.2 Hz, 2H), 1.35 (m, 2H), 0.84 (s, 3H), 0.75 (t, *J* = 7.6 Hz, 3H); LCMS calculated for $C_{24}H_{30}N_6O_3$: m/z = 450; found: m/z = 451 $(M+H)^+$.

_N_-(1_H_-indazol-7-yl)-4-(3-(2-methyl-2-morpholinobutyl)ureido)benzamide (BB581)

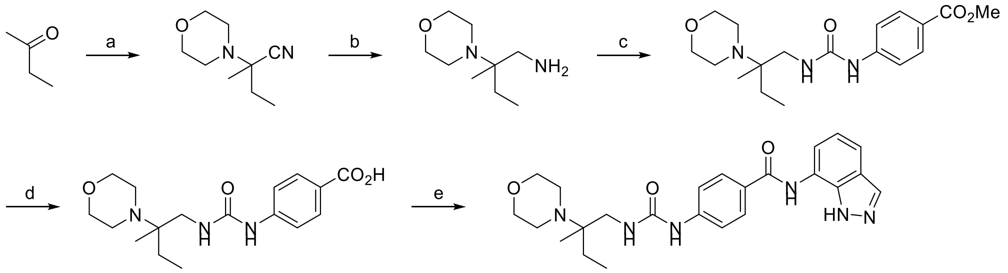

BB0581

Reagents and conditions: (a) morpholine, 2-hydroxy-2-methylpropanenitrile, $MgSO_4$, DMA, 45 °C, 12 h, 27% yield; (b) $LiAlH_4$, THF, 0 °C, 12 h, 26% yield; (c) methyl 4-aminobenzoate, (4-nitrophenyl) carbonochloridate, THF, RT, 12 h, 59% yield; (d) $LiOH.H_2O$, THF, 60 °C, 12 h, 30% yield; (e) 1*H*-indazol-7-amine, DIEA, HATU, DMF, 80 °C, 1 h, 37% yield.

Step a: 2-methyl-2-morpholinobutanenitrile

To a solution of morpholine (18.1 g, 208 mmol, 1.5 eq), butan-2-one (10.0 g, 138.69 mmol, 1.0 eq), and 2-hydroxy-2-methylpropanenitrile (11.8 g, 138.69 mmol, 1.0 eq) in DMA (20 mL) at RT under N_2(g) was added $MgSO_4$ (33.4 g, 277.37 mmol, 2.0 eq). The resulting reaction mixture was stirred at 45 °C for 12 h. The reaction mixture was poured into H_2O (100 mL) and extracted with EtOAc (3 x 100 mL). The combined organic phase was dried over anhydrous Na_2SO_4 and filtered, and the filtrate was concentrated under reduced pressure. The residue was purified by silica gel column chromatography (PE:EtOAc = 10:1 to 3:1) to afford 2-methyl-2-morpholinobutanenitrile (6.3 g, 27% yield) as a colorless oil. ^{1}H NMR (400 MHz, $CDCl_3$) δ 3.68 (t, *J* = 4.8 Hz, 4H), 2.55 (t, *J* = 4.4 Hz, 4H), 1.75 (q, *J* = 7.2 Hz, 2H), 1.36 (s, 3H), 0.93 (t, *J* = 7.6 Hz, 3H); LCMS calculated for $C_9H_{16}N_2O$: m/z = 168; found: m/z = 169 $(M+H)^+$.

Step b: 2-methyl-2-morpholinobutan-1-amine

To a solution of 2-methyl-2-morpholinobutanenitrile (3.0 g, 17.83 mmol, 1.0 eq) in THF (40 mL) at 0 °C

under N_2(g) was added $LiAlH_4$ (2.36 g, 62.23 mmol, 3.49 eq). The resulting reaction mixture was stirred at RT for 12 h. The reaction mixture was combined with another same scale batch reaction. Then the combined mixture was poured into H_2O (50 mL) at 0 °C and extracted with EtOAc (3 x 50 mL). The combined organic phase was dried over anhydrous Na_2SO_4 and filtered, and the filtrate was concentrated under reduced pressure to afford 2-methyl-2-morpholinobutan-1-amine (1.6 g, 26% yield) as a white solid. ^{1}H NMR (400 MHz, $CDCl_3$) δ 3.62 (m, 4H), 2.47 (m, 5H), 2.33 (m, 1H), 1.50 (m, 1H), 1.20 (m, 1H), 0.91 (d, *J* = 6.4 Hz, 3H), 0.83 (m, 3H); LCMS calculated for $C_9H_{20}N_2O$: m/z = 172; found: m/z = 173 $(M+H)^+$.

Step c: methyl 4-(3-(2-methyl-2-morpholinobutyl)ureido)benzoate

To a solution of methyl 4-aminobenzoate (219 mg, 1.45 mmol, 1.0 eq) in THF (5 mL) at RT under N_2(g) was added (4-nitrophenyl) carbonochloridate (292 mg, 1.45 mmol, 1.0 eq). The resulting reaction mixture was stirred at RT for 0.5 h. Then 2-methyl-2-morpholinobutan-1-amine (500 mg, 2.90 mmol, 2.0 eq) was added, and the mixture was stirred at RT for 11.5 h. The reaction mixture was concentrated under reduced pressure. The crude product was purified by silica gel column chromatography (PE:EtOAc = 10:1 to 3:1) to afford methyl 4-(3-(2-methyl-2-morpholinobutyl)ureido)benzoate (300 mg, 59%) as a yellow solid. LCMS calculated for $C_{18}H_{27}N_3O_4$: m/z = 349; found: m/z = 350 $(M+H)^+$.

Step d: 4-(3-(2-methyl-2-morpholinobutyl)ureido)benzoic acid

To a solution of methyl 4-(3-(2-methyl-2-morpholinobutyl)ureido)benzoate (400 mg, 1.14 mmol, 1.0 eq) in THF (32 mL) and H_2O (8 mL) at RT under N_2(g) was added $LiOH.H_2O$ (480 mg, 11.45 mmol, 10.0 eq). The resulting reaction mixture was stirred at 60 °C for 12 h. The reaction mixture was filtered, and the filtrate was concentrated under reduced pressure. The crude product was purified by reverse-phase preparative HPLC (column: Phenomenex luna C18 250*50mm*10µm; mobile phase: [water(0.04%HCl)-ACN]; B%: 1%-30%,10 min) to afford 4-(3-(2-methyl-2-morpholinobutyl)ureido)benzoic acid (120 mg, 30% yield) as a white solid. ^{1}H NMR (400 MHz, DMSO-d_6) δ 12.58 (br, s, 1H), 9.55 (m, 2H), 7.85 (d, *J* = 8.8 Hz, 2H), 7.52 (d, *J* = 8.8 Hz, 2H), 7.17 (m, 1H), 3.93 (m, 4H), 3.60 (m, 5H), 3.54 (m, 1H), 3.21 (m, 2H), 1.80 (m, 2H), 1.29 (s, 3H), 0.99 (t, *J* = 7.2 Hz, 3H); LCMS calculated for $C_{17}H_{25}N_3O_4$: m/z = 335; found: m/z = 336 $(M+H)^+$.

Step e: *N*-(1*H*-indazol-7-yl)-4-(3-(2-methyl-2-morpholinobutyl)ureido)benzamide (BB581)

To a solution of 4-(3-(2-methyl-2-morpholinobutyl)ureido)benzoic acid (50 mg, 149 µmol, 1.0 eq) and 1*H*-indazol-7-amine (30 mg, 223 µmol, 1.5 eq) in DMF (1 mL) at RT under N_2(g) was added DIEA (48 mg, 373 µmol, 2.5 eq) and HATU (85 mg, 223 µmol, 1.5 eq). The resulting reaction mixture was stirred at 80 °C for 12 h. The reaction mixture was combined with another same scale batch reaction. Then the combined mixture was filtered, and the filtrate was purified by reverse-phase preparative HPLC (column: Waters Xbridge BEH C18 100*30mm*10µm; mobile phase: [water(10mM NH_4HCO_3)-ACN]; B%: 15%-45%,10 min) to afford *N*-(1*H*-indazol-7-yl)-4-(3-(2-methyl-2-morpholinobutyl)ureido)benzamide (50.1 mg, 37% yield, 100% purity by LCMS) as a white solid. ^{1}H NMR (400 MHz, CD_3OD) δ 8.11 (s, 1H), 8.01 (d, *J* = 8.4 Hz, 2H), 7.68 (d, *J* = 8.0 Hz, 1H), 7.59 (d, *J* = 8.8 Hz, 2H), 7.50 (d, *J* = 7.2 Hz, 1H), 7.19 (t, *J* = 7.6 Hz, 1H), 3.74 (t, *J* = 4.4 Hz, 4H), 3.26 (s, 2H), 2.65 (m, 4H), 1.56 (m, 2H), 1.06 (s, 3H), 0.95 (t, *J* = 7.6 Hz, 3H); LCMS calculated for $C_{24}H_{30}N_6O_3$: m/z = 450; found: m/z = 451 $(M+H)^+$.

***N*-(4-(3-(pyrazin-2-ylmethyl)ureido)phenyl)-1*H*-indazole-7-carboxamide (BB582)**

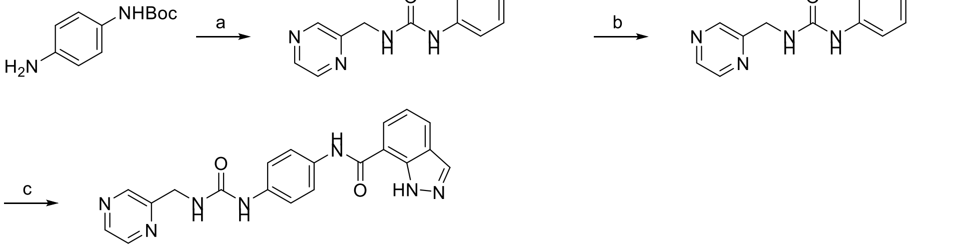

BB0582

Reagents and conditions: (a) triphosgene, pyrazin-2-ylmethanamine, TEA, DCM, °0 C, 2 h, 40% yield; (b) HCl/MeOH, RT, 1 h, crude; (c) 1*H*-indazole-7-carboxylic acid, DIEA, HATU, DMF, RT, 1 h, 12% yield for 2 steps.

Step a: *tert*-butyl (4-(3-(pyrazin-2-ylmethyl)ureido)phenyl)carbamate

To a solution of triphosgene (940 mg, 3.17 mmol, 0.3 eq) in DCM (10 mL) at 0 °C under N_2(g) was added a solution of *tert*-butyl *N*-(4-aminophenyl)carbamate (2.0 g, 9.60 mmol, 1.0 eq) in DCM (20 mL). The resulting reaction mixture was stirred at 0 °C for 1 h. Then pyrazin-2-ylmethanamine (1.05 g, 9.60 mmol, 1.0 eq) and TEA (1.94 g, 19.21 mmol, 2.0 eq) were added, and the reaction mixture was stirred at 0 °C for 1 h. The reaction mixture was poured into H_2O (10 mL) and extracted with EtOAc/THF (1:1, 3 x 100 mL). The combined organic phase was dried over anhydrous Na_2SO_4 and filtered, and the filtrate was concentrated under reduced pressure. The crude product was triturated with EtOAc (20 mL) at RT for 10 min. The resulting slurry was filtered and the solid was collected and dried under reduced pressure to afford *tert*-butyl *N*-[4-(pyrazin-2-ylmethylcarbamoylamino)phenyl]carbamate (1.7 g, 40% yield, 78% purity by LCMS) as a black solid. ^{1}H NMR (400 MHz, DMSO-d_6) δ 9.12 (m, 1H), 8.60 (m, 2H), 8.53 (m, 1H), 7.30 (m, 4H), 6.72 (t, *J* = 5.6 Hz, 1H), 4.45 (d, *J* = 5.6 Hz, 2H), 1.46 (m, 9H); LCMS calculated for $C_{17}H_{21}N_5O_3$: m/z = 343; found: m/z = 344 $(M+H)^+$

Step b: 1-(4-aminophenyl)-3-(pyrazin-2-ylmethyl)urea

To a solution of HCl/MeOH (4 M, 0.15 mL, 1.0 eq) at RT under N_2(g) was added *tert*-butyl *N*-[4-(pyrazin-2-ylmethylcarbamoylamino)phenyl]carbamate (0.2 g, 0.58 mmol, 1.0 eq). The resulting reaction mixture was stirred at RT for 1 h. The reaction mixture was concentrated under reduced pressure to afford crude 1-(4-aminophenyl)-3-(pyrazin-2-ylmethyl)urea (0.3 g, crude) as a yellow solid which was used without further purification. LCMS calculated for $C_{12}H_{13}N_5O$: m/z = 243; found: m/z = 266 $(M+Na)^+$.

Step c: *N*-(4-(3-(pyrazin-2-ylmethyl)ureido)phenyl)-1*H*-indazole-7-carboxamide (BB582)

To a mixture of 1-(4-aminophenyl)-3-(pyrazin-2-ylmethyl)urea (0.3 g, 1.23 mmol, 1.0 eq) and 1*H*-indazole-7-carboxylic acid (300 mg, 1.85 mmol, 1.5 eq) in DMF (5 mL) at RT under N_2(g) was added DIEA (398 mg, 3.08 mmol, 2.5 eq) and HATU (703 mg, 1.85 mmol, 1.5 eq). The resulting reaction mixture was stirred at RT for 1 h. The reaction mixture was poured into H_2O (10 mL) and extracted with EtOAc (2 x 20 mL). The combined organic phase was dried over anhydrous Na_2SO_4 and filtered, and the filtrate was concentrated under reduced pressure to give a residue. The residue was purified by reverse-phase preparative HPLC (column: Welch Xtimate C18 100*25mm*3μm; mobile phase: [water(0.05%HCl)-ACN]; B%: 5%-35%, 8 min) to afford *N*-[4-(pyrazin-2-ylmethylcarbamoylamino)phenyl]-1*H*-indazole-7-carboxamide (61 mg, 12% yield, 96% by LCMS purity) as a yellow solid. ^{1}H NMR (400 MHz, DMSO-d_6) δ 13.16 (s, br, 1H), 10.28 (s, br, 1H), 8.76 (s, 1H), 8.65 (s, 1H), 8.61 (s, 1H), 8.55 (s, 1H), 8.18 (s, 1H), 8.05 (d, *J* = 7.2 Hz, 1H), 8.01 (d, *J* = 8 Hz, 1H), 7.68 (d, *J* = 8.8 Hz, 2H), 7.41 (d, *J* = 8.8 Hz, 2H), 6.80 (m, 1H), 6.78 (m, 1H), 4.48 (d, *J* = 5.2 Hz, 2H); LCMS calculated for $C_{20}H_{17}N_7O_2$: m/z = 387; found: m/z = 388 $(M+H)^+$.

N-(4-(3-benzylureido)phenyl)-1H-indazole-7-carboxamide (BB583)

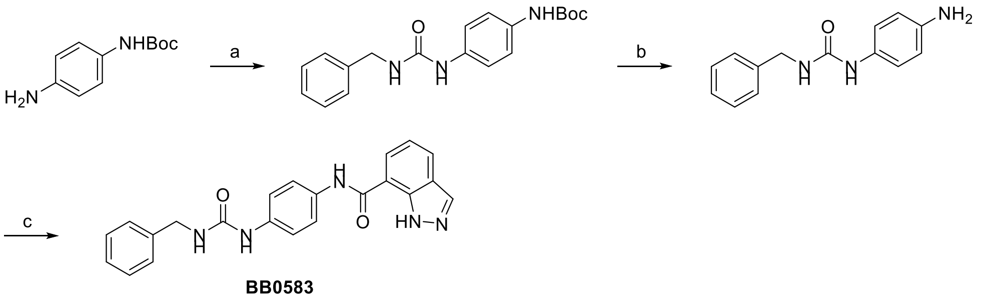

BB0583

Reagents and conditions: (a) triphosgene, phenylmethanamine, TEA, DCM, 0 °C, 2 h, 61% yield; (b) TFA, DCM, RT, 1 h, crude; (c) 1*H*-indazole-7-carboxylic acid, DIEA, HATU, DMF, RT, 1 h, 15% yield for 2 steps.

Step a: *tert*-butyl (4-(3-benzylureido)phenyl)carbamate

To a mixture of triphosgene (940 mg, 3.17 mmol, 0.3 eq) in DCM (10 mL) at 0 °C was added a mixture of *tert*-butyl (4-aminophenyl)carbamate (2.0 g, 9.60 mmol, 1.0 eq) in DCM (20 mL). The resulting reaction mixture was stirred for 1 h. Then a mixture of phenylmethanamine (1.03 g, 9.60 mmol, 1.0 eq) and TEA (1.94 g, 19.21 mmol, 2.0 eq) was added at 0 °C, and the reaction mixture was stirred at 0 °C for 1 h. The reaction mixture was poured into H_2O (20 mL) and extracted with EtOAc/THF (1:1, 3 x 30 mL). The combined organic phase was dried over anhydrous Na_2SO_4 and filtered, and the filtrate was concentrated under vacuum. The residue was purified by silica gel column chromatography (PE:EtOAc = 10:1 to 0:1) to afford *tert*-butyl (4-(3-benzylureido)phenyl)carbamate (2.0 g, 61% yield, 64% purity by LCMS) as a white solid. ^{1}H NMR (400 MHz, DMSO-d_6) δ 9.15 (m, 1H), 8.41 (m, 1H), 7.31 (m, 9H), 6.48 (m, 1H), 4.27 (m, 2H), 1.47 (m, 9H); LCMS calculated for $C_{19}H_{23}N_3O_3$: m/z = 341; found: m/z = 342 $(M+H)^+$.

Step b: 1-(4-aminophenyl)-3-benzylurea

To a solution of *tert*-butyl (4-(3-benzylureido)phenyl)carbamate (500 mg, 1.46 mmol, 1.0 eq) in DCM (12.5 mL) at RT was added TFA (3.85 g, 33.77 mmol, 23.0 eq). The resulting reaction mixture was stirred for 1 h. The reaction mixture was concentrated under reduced pressure to afford crude 1-(4-aminophenyl)-3-benzylurea (220 mg, crude) as a yellow solid which was used without further purification. LCMS calculated for $C_{14}H_{15}N_3O$: m/z = 241; found: m/z = 242 $(M+H)^+$.

Step c: *N*-(4-(3-benzylureido)phenyl)-1*H*-indazole-7-carboxamide (BB583)

To a mixture of 1-(4-aminophenyl)-3-benzylurea (170 mg, 0.70 mmol, 1.0 eq) and 1*H*-indazole-7-carboxylic acid (171 mg, 1.06 mmol, 1.5 eq) in DMF (4 mL) at RT was added DIEA (228 mg, 1.76 mmol, 2.5 eq) and HATU (402 mg, 1.06 mmol, 1.5 eq). The resulting reaction mixture was stirred for 1 h. The reaction mixture was poured into H_2O (20 mL) and extracted with EtOAc (2 x 20 mL). The combined organic phase was dried over anhydrous Na_2SO_4 and filtered, and the filtrate was concentrated under reduced pressure to give a residue. The residue was purified by reverse-phase preparative HPLC (column: Phenomenex Luna C18 75*30mm*3μm; mobile phase: [water(0.2%TFA)-MeOH]; B%: 40%-70%, 8 min) to afford *N*-(4-(3-benzylureido)phenyl)-1*H*-indazole-7-carboxamide (41.6 mg, 15.16% yield, 98.97% by LCMS purity) as a white solid. ^{1}H NMR (400 MHz, DMSO-d_6) δ 13.18 (s, br, 1H), 10.29 (s, br, 1H), 8.55 (s, 1H), 8.20 (s, 1H), 8.11 (d, *J* = 8 Hz, 1H), 8.01 (d, *J* = 8 Hz, 1H), 7.68 (d, *J* = 8.8 Hz, 2H), 7.42 (d, *J* = 8.8 Hz, 2H), 7.34 (m, 4H), 7.27 (d, *J* = 8 Hz, 2H), 6.61 (t, *J* = 6 Hz, 1H), 4.31 (d, *J* = 6 Hz, 2H); LCMS calculated for $C_{22}H_{19}N_5O_2$: m/z = 385; found: m/z = 386 $(M+H)^+$.

N-(4-(3-neopentylureido)phenyl)-1*H*-indazole-7-carboxamide (BB584)

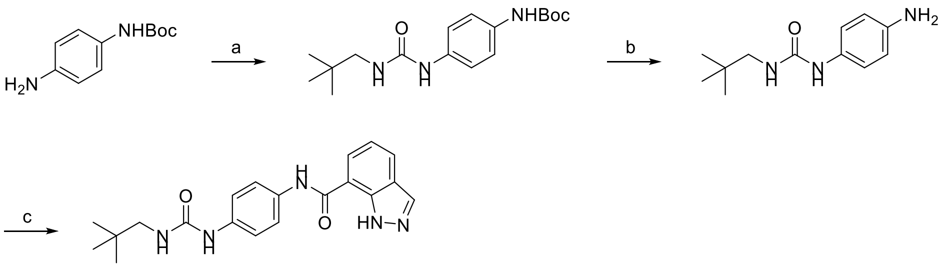

BB0584

Reagents and conditions: (a) 2,2-dimethylpropan-1-amine, triphosgene, TEA, DCM, 0 °C, 10 min; (b) TFA, DCM, RT, 1 h, crude; (c) 1*H*-indazole-7-carboxylic acid, DIEA, HATU, DMF, RT, 1 h, 45% yield for 2 steps.

Step a: *tert*-butyl (4-(3-neopentylureido)phenyl)carbamate

To a solution of triphosgene (940 mg, 3.17 mmol, 0.3 eq) in DCM (10 mL) at 0 °C under N_2(g) was added a solution of *tert*-butyl *N*-(4-aminophenyl)carbamate (2.0 g, 9.60 mmol, 1.0 eq) in DCM (20 mL). The resulting reaction mixture was stirred at 0 °C for 10 min. Then 2,2-dimethylpropan-1-amine (837 mg, 9.60 mmol, 1.0 eq) and TEA (1.9 g, 19.21 mmol, 2.0 eq) were added, and the mixture was stirred at 0 °C for 1 h. The reaction mixture was poured into H_2O (20 mL) and extracted with EtOAc/THF (1:1, 3 x 30 mL). The combined organic phase was dried over anhydrous Na_2SO_4 and filtered, and the filtrate was concentrated under reduced pressure to give a residue. The residue was purified by silica gel column chromatography (PE:EtOAc = 10:1 to 0:1) to afford *tert*-butyl *N*-[4-(2,2-dimethylpropylcarbamoylamino)phenyl]carbamate (2.0 g, 65% yield) as a white solid. ^{1}H NMR (400 MHz, DMSO-d_6) δ 9.15 (m, 1H), 8.33 (m, 1H), 7.28 (m, 4H), 5.90 (m, 1H), 2.90 (d, *J* = 6.8 Hz, 1H), 2.82 (d, *J* = 6.8 Hz, 1H), 1.47 (s, 2H), 0.84 (s, 9H); LCMS calculated for $C_{17}H_{27}N_3O_3$: m/z = 321; found: m/z = 322 $(M+H)^+$.

Step b: 1-(4-aminophenyl)-3-(2-morpholinobutyl)urea

To a solution of TFA (2.50 mL) in DCM (12.5 mL) at RT under N_2(g) was added *tert*-butyl *N*-[4-(2,2-dimethylpropylcarbamoylamino)phenyl]carbamate (500 mg, 1.56 mmol, 1.0 eq). The resulting reaction mixture was stirred at RT for 1 h. The reaction mixture was concentrated under reduced pressure to afford 1-(4-aminophenyl)-3-(2,2-dimethylpropyl)urea (300 mg, crude, 50% purity by LCMS) was as a yellow solid which was used without further purification. LCMS calculated for $C_{12}H_{19}N_3O$: m/z = 221; found: m/z = 222 $(M+H)^+$.

Step c: *N*-(4-(3-(2-morpholinobutyl)ureido)phenyl)-1*H*-indazole-7-carboxamide (BB584)

To a solution of 1-(4-aminophenyl)-3-(2,2-dimethylpropyl)urea (250 mg, 1.13 mmol, 1.0 eq) and 1*H*-indazole-7-carboxylic acid (274 mg, 1.69 mmol, 1.5 eq) in DMF (5 mL) at RT under N_2(g) was added DIEA (365 mg, 2.82 mmol, 2.5 eq) and HATU (644 mg, 1.69 mmol, 1.5 eq). The resulting reaction mixture was stirred at RT for 1 h. The reaction mixture was poured into H_2O (20 mL) and extracted with EtOAc (2 x 20 mL). The combined organic phase was dried over anhydrous Na_2SO_4, filtered, and the filtrate was concentrated under reduced pressure to give a residue. The residue was purified by reverse-phase preparative HPLC (column: Phenomenex Gemini-NX 150*30mm*5μm; mobile phase: [water(0.2%FA)-ACN]; B%: 30%-65%, 8 min) to afford *N*-[4-(2,2-dimethylpropylcarbamoylamino)phenyl]-1*H*-indazole-7-carboxamide (187.7 mg, 45% yield, 99.88% purity by LCMS) as a white solid. ^{1}H NMR (400 MHz, CD_3OD) δ 13.17 (s, br, 1H), 10.26 (s, br, 1H), 8.36 (s, 1H), 8.16 (s, 1H), 8.00 (m, 2H), 7.65 (d, *J* = 8.8 Hz, 2H), 7.37 (d, *J* = 8.8 Hz, 2H), 7.24 (d, *J* = 7.6 Hz, 1H), 6.13 (t, *J* = 6.4 Hz, 1H), 2.90 (d, *J* = 6, 2H), 0.85 (s, 9H); LCMS calculated for $C_{20}H_{23}N_5O_2$: m/z = 365; found: m/z = 366 $(M+H)^+$.

***N*-[5-[(2-methyl-2-morpholino-butyl)carbamoylamino]thiazol-2-yl]-1*H*-indazole-7-carboxamide (BB585)**

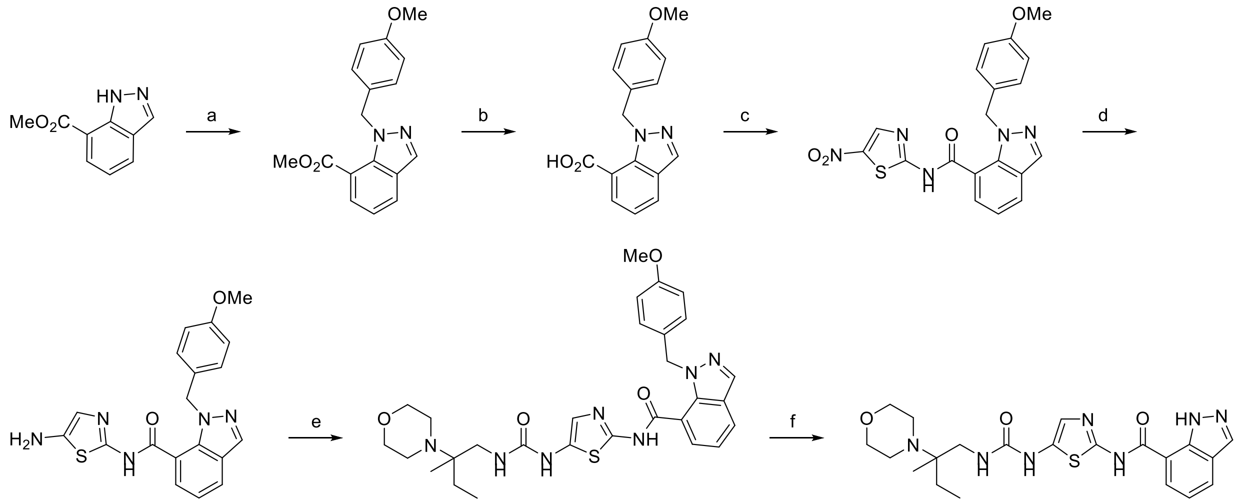

BB0585

Reagents and conditions: (a) NaH, PMB-Cl, DMF, 0 °C-RT,12.5 h, 65.40% yield; (b) LiOH.H_2O, MeOH, H_2O, RT, 12 h, crude; (c) 5-nitrothiazol-2-amine, BOP, DIEA, DMF, RT, 12 h, 70.72% yield; (d) Pd/C, H_2 (15 psi), MeOH, RT, 12 h, crude; (e) 2-methyl-2-morpholino-butan-1-amine, bis(2,5-dioxopyrrolidin-1-yl)carbonate, pyridine, MeCN, RT, 14 h, 42% yield; (f) TFA, 100 °C, 30 min, microwave, 20% yield.

Step a: Methyl 1-[(4-methoxyphenyl)methyl]indazole-7-carboxylate

To a solution of methyl 1*H*-indazole-7-carboxylate (2 g, 11.35 mmol, 1 eq) in DMF (10 mL) was added NaH (60% in mineral oil, 590.33 mg, 14.76 mmol, 1.3 eq) at 0 °C. The reaction was stirred at 0 °C for 0.5 h, then PMB-Cl (1.78 g, 11.35 mmol, 1.55 mL, 1 eq) was added. The solution was stirred at RT for 12 h, then was poured into water (60 mL) and extracted with ethyl acetate (3 x 40 mL). The combined organic phases were washed with brine (50 mL), dried over anhydrous Na_2SO_4, filtered, and concentrated in vacuum. The residue was purified by flash silica gel chromatography (ISCO®; 20 g SepaFlash® silica flash column, 30-100% ethyl acetate/petroleum ether gradient at 60 mL/min) to give methyl 1-[(4-methoxyphenyl)methyl]indazole-7-carboxylate (2.2 g, 7.42 mmol, 65.40% yield) as a yellow oil.

Step b: 1-[(4-methoxyphenyl)methyl]indazole-7-carboxylic acid

LiOH.H_2O (404.99 mg, 9.65 mmol, 1.3 eq) was added to a solution of methyl 1-[(4-methoxyphenyl)methyl]indazole-7-carboxylate (2.2 g, 7.42 mmol, 1 eq) in MeOH (10 mL) and H_2O (5 mL) at RT, then the reaction was stirred at RT for 12 h. The solution was concentrated under reduced pressure to remove the solvent. The residue was diluted with H_2O (10 mL), and the pH value of the solution was adjusted to 4 with HCl (2M). Some solid precipitated, and the mixture was filtered. The filter cake was collected and dried in vacuum to give 1-[(4-methoxyphenyl)methyl]indazole-7-carboxylic acid (1.5 g, crude) as a yellow solid. LCMS calculated for $C_{16}H_{14}N_2O_3$: m/z = 282; found: m/z = 281 $(M-1)^-$. This material was used in the next step without further purification.

Step c: 1-[(4-methoxyphenyl)methyl]-*N*-(5-nitrothiazol-2-yl)indazole-7-carboxamide

To a solution of 1-[(4-methoxyphenyl)methyl]indazole-7-carboxylic acid (0.78 g, 2.76 mmol, 1 eq) in DMF (5 mL) was added 5-nitrothiazol-2-amine (481.24 mg, 3.32 mmol, 1.2 eq), BOP (1.34 g, 3.04 mmol, 1.1 eq), and DIEA (892.77 mg, 6.91 mmol, 1.20 mL, 2.5 eq). The suspension was stirred at RT for 12 h then was filtered. The filter cake was washed with MeOH (5 mL) and dried under reduced pressure to give 1-[(4-methoxyphenyl)methyl]-*N*-(5-nitrothiazol-2-yl)indazole-7-carboxamide (0.8 g, 1.95 mmol, 70.72% yield) as a yellow solid. ^1H NMR (400 MHz, DMSO-d_6) δ 13.01 (br s, 1H), 8.83 (s, 1H), 8.75 (s, 1H), 8.21 (m, 2H), 7.43 (d, *J* = 8.4 Hz, 2H), 7.34 (dd, *J* = 8.8, 7.6 Hz, 1H), 6.95 (d, *J* = 8.8 Hz, 2H), 5.75 (s, 2H), 3.73 (s, 3H). This material was used in the next step without further purification.

Step d: *N*-(5-aminothiazol-2-yl)-1-[(4-methoxyphenyl)methyl]indazole-7-carboxamide

To a solution of 1-[(4-methoxyphenyl)methyl]-*N*-(5-nitrothiazol-2-yl)indazole-7-carboxamide (1 g, 2.44 mmol, 1 eq) in MeOH (150 mL) was added 10% Pd/C (1 g) under N_2. The suspension was degassed under vacuum and purged with H_2 several times. The suspension was stirred under H_2 (15 psi) at RT for 12 h. The suspension was filtered, and the filtrate was concentrated under reduced pressure to give *N*-(5-aminothiazol-2-yl)-1-[(4-methoxyphenyl)methyl]indazole-7-carboxamide (0.26 g, 685.23 μmol) as a yellowish solid. The crude product was used in the next step without further purification.

Step e: 1-[(4-methoxyphenyl)methyl]-*N*-[5-[(2-methyl-2-morpholino-butyl)carbamoylamino]thiazol-2-yl]indazole-7-carboxamide

To a solution of *N*-(5-aminothiazol-2-yl)-1-[(4-methoxyphenyl)methyl]indazole-7-carboxamide (230 mg, 606.16 μmol, 1 eq) in MeCN (5 mL) was added pyridine (47.95 mg, 606.16 μmol, 48.93 μL, 1 eq) and bis(2,5-dioxopyrrolidin-1-yl) carbonate (155.28 mg, 606.16 μmol, 1 eq) in one portion under N_2. The solution was stirred at 20 °C for 2 h, then 2-methyl-2-morpholino-butan-1-amine (125.31 mg, 727.40 μmol, 1.2 eq) was added, and the reaction was stirred at 20 °C for 12 h. The solution was poured into water (20 mL) and extracted with ethyl acetate (2 x 15 mL). The combined organic phases were washed with brine (10 mL), dried over anhydrous Na_2SO_4, filtered, and concentrated in vacuum. The residue was purified by flash silica gel chromatography (ISCO®; 4 g SepaFlash® silica flash column, 0-100% ethyl acetate/petroleum ether gradient at 50 mL/min) to afford 1-[(4-methoxyphenyl)methyl]-*N*-[5-[(2-methyl-2-morpholino-butyl)carbamoylamino]thiazol-2-yl]indazole-7-carboxamide (160 mg, 46% yield) as a yellow solid. LCMS calculated for $C_{29}H_{35}N_7O_4S$: m/z = 577; found: m/z = 578 $(M+H)^+$.

Step f: *N*-[5-[(2-methyl-2-morpholino-butyl)carbamoylamino]thiazol-2-yl]-1*H*-indazole-7-carboxamide (BB585)

1-[(4-methoxyphenyl)methyl]-*N*-[5-[(2-methyl-2-morpholino-butyl)carbamoylamino]thiazol-2-l]indazole-7-carboxamide (140 mg, 242.34 μmol, 1 eq) was taken up in TFA (1.5 mL) in a microwave tube. The tube was sealed and heated at 100 °C for 30 min under microwave. The reaction solution was concentrated under reduced pressure to remove the solvent. The residue was purified by prep-HPLC (HCl condition: column: Phenomenex Luna C18 80*40mm*3μm; mobile phase: [water(HCl)-ACN]; B%: 6%-25%,7 min) to afford *N*-[5-[(2-methyl-2-morpholinobutyl)carbamoylamino]thiazol-2-yl]-1*H*-indazole-7-carboxamide (26.3 mg, 95.6% purity, HCl salt) as an off-white solid. ^1H NMR (400 MHz, CD_3OD) δ 8.28 (s, 1H), 8.19 (br d, *J* = 7.2 Hz, 1H), 8.17 (br d, *J* = 8 Hz, 1H), 7.36 (t, *J* = 8 Hz, 1H), 7.27 (s, 1H), 4.14 (m, 2H), 3.91 (br t, *J* = 12 Hz, 2H), 3.64 (m, 4H), 3.35 (br s, 1H), 3.25 (s, 1H), 1.87 (m, 2H), 1.40 (s, 3H), 1.09 (t, *J* = 7.2 Hz, 3H); ^1H NMR (400 MHz, DMSO-d_6) δ 13.18 (m, 1H), 9.78 (s, 1H), 9.41 (m, 1H), 8.25 (s, 2H), 8.05 (d, *J* = 7.6 Hz, 1H), 7.25 (t, *J* = 7.6 Hz, 1H), 7.09 (s, 1H), 6.99 (m, 1H), 4.01 (m, 2H), 3.87 (m, 2H), 3.59 (m, 4H), 3.22 (m, 2H), 1.78 (m, 2H), 1.28 (s, 3H), 0.98 (t, *J* = 7.6 Hz, 3H); LCMS calculated for $C_{21}H_{27}N_7O_3S$: m/z = 457; found: m/z = 458 $(M+H)^+$.

***N*-[5-[(2-methyl-2-morpholino-butyl)carbamoylamino]-1*H*-pyrazol-3-yl]-1*H*-indazole-7-carboxamide (BB586)**

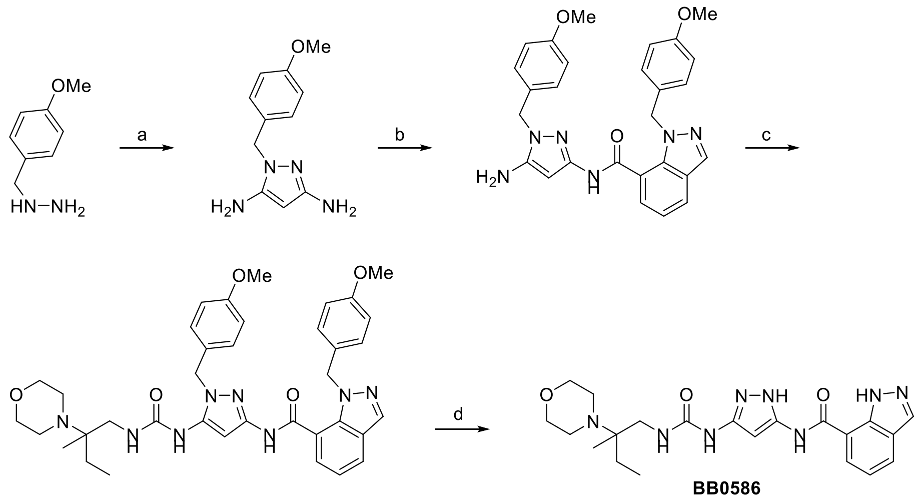

Reagents and conditions: (a) propanedinitrile, EtOH, TEA, 80 °C, 12 h, crude; (b) 1-[(4-methoxyphenyl) methyl]indazole-7-carboxylic acid, HATU, TEA, DMF, RT, 12 h, 22.52% yield; (c) 2-methyl-2-morpholino-butan-1-amine, bis(2,5-dioxopyrrolidin-1-yl)carbonate, pyridine, MeCN, RT,14 h, 19.49%; (d) TFA, 85 °C, 2 h, 11.93% yield

Step a: 1-[(4-methoxyphenyl)methyl]pyrazole-3,5-diamine

To a solution of (4-methoxyphenyl)methylhydrazine hydrochloride (2.86 g, 15.14 mmol, 1 eq) and propanedinitrile (1 g, 15.14 mmol, 952.38 µL, 1 eq) in EtOH (15 mL) was added TEA (1.23 g, 12.11 mmol, 1.69 mL, 0.8 eq), then the solution was stirred at 80 °C for 12 h under N_2 atmosphere. The solution was concentrated under reduced pressure to remove solvent. The residue was diluted with water (100 mL) and extracted with ethyl acetate (2 x 50 mL). The combined organic layers were washed with brine (100 mL), dried over Na_2SO_4, filtered, and concentrated under reduced pressure to give a residue. The residue was purified by flash silica gel chromatography (ISCO®; 20 g SepaFlash® silica flash column, 0-100% ethyl acetate/petroleum ether gradient at 80 mL/min) to afford 1-[(4-methoxyphenyl)methyl]pyrazole-3,5-diamine (3 g, crude) as brown oil. This material was used in the next step without further purification.

Step b: *N*-[5-amino-2-[(4-methoxyphenyl)methyl]pyrazol-3-yl]-1-[(4-methoxyphenyl)methyl]indazole-7-carboxamide

A suspension of 1-[(4-methoxyphenyl)methyl]indazole-7-carboxylic acid (1 g, 3.54 mmol, 1 eq), 1-[(4-methoxyphenyl)methyl]pyrazole-3,5-diamine (850.46 mg, 3.90 mmol, 1.1 eq), HATU (1.62 g, 4.25 mmol, 1.2 eq) and TEA (1.08 g, 10.63 mmol, 1.48 mL, 3 eq) in DMF (10 mL) was degassed and purged with N_2 3 times, then the suspension was stirred at RT for 12 h under N_2 atmosphere. The solution was poured into water (50 mL) and extracted with ethyl acetate (3 x 30 mL). The combined organic phases were washed with brine (50 mL), dried over anhydrous Na_2SO_4, filtered, and concentrated in vacuum. The residue was purified by flash silica gel chromatography (ISCO®; 20 g SepaFlash® silica flash column, 0-100% ethyl acetate/petroleum ether gradient at 80 mL/min) to afford *N*-[5-amino-2-[(4-methoxyphenyl)methyl]pyrazol-3-yl]-1-[(4-methoxyphenyl)methyl]indazole-7-carboxamide (700 mg, 797.87 umol, 22.52% yield, 55% purity) as a brown oil.

Step c: 1-[(4-methoxyphenyl)methyl]-*N*-[1-[(4-methoxyphenyl)methyl]-5-[(2-methyl-2-morpholino-butyl)carbamoylamino]pyrazol-3-yl]indazole-7-carboxamide

To a solution of *N*-[5-amino-1-[(4-methoxyphenyl)methyl]pyrazol-3-yl]-1-[(4-methoxyphenyl)methyl]indazole-7-carboxamide (0.4 g, 828.96 µmol, 1 eq) in MeCN (1 mL) was added pyridine (65.57 mg, 828.96 µmol, 66.91 µL, 1 eq) and bis(2,5-dioxopyrrolidin-1-yl) carbonate (212.35 mg, 828.96 µmol, 1 eq) in one portion under N_2. The solution was stirred at RT for 2 h, then 2-methyl-2-

morpholino-butan-1-amine (171.36 mg, 994.75 µmol, 1.2 eq) was added, and the reaction was stirred at RT for 12 h. The solution was poured into water (20 mL) and extracted with ethyl acetate (2 x 15 mL). The combined organic phases were washed with brine (10 mL), dried over anhydrous Na_2SO_4, filtered, and concentrated in vacuum. The residue was purified by flash silica gel chromatography (ISCO®; 4 g SepaFlash® silica flash column, 0-100% ethyl acetate/petroleum ether gradient at 50 mL/min) to afford 1-[(4-methoxyphenyl)methyl]-*N*-[1-[(4-methoxyphenyl)methyl]-5-[(2-methyl-2-morpholino-butyl)carbamoylamino]pyrazol-3-yl]indazole-7-carboxamide (110 mg, 161.58 µmol, 19.49 % yield) as a yellow solid.

Step d: *N*-[5-[(2-methyl-2-morpholino-butyl)carbamoylamino]-1*H*-pyrazol-3-yl]-1*H*-indazole-7-carboxamide (BB586)

1-[(4-Methoxyphenyl)methyl]-*N*-[1-[(4-methoxyphenyl)methyl]-5-[(2-methyl-2-morpholino-butyl)carbamoylamino]pyrazol-3-yl]indazole-7-carboxamide (200 mg, 293.77 µmol, 1 eq) was taken up in TFA (2 mL), and the solution was stirred at 85 °C for 2 h. The reaction solution was concentrated under reduced pressure to remove the solvent. The residue was purified by prep-HPLC (HCl condition: column: Phenomenex Luna C18 80*40mm*3µm; mobile phase: [water(HCl)-ACN]; B%: 10%-26%,7 min) to afford *N*-[5-[(2-methyl-2-morpholino-butyl)carbamoylamino]-1*H*-pyrazol-3-yl]-1*H*-indazole-7-carboxamide (50.0 mg, 99.16% purity, HCl) as an off-white solid. ^{1}H NMR (400 MHz, DMSO-d_6) δ 12.98 (m, 1H), 11.28 (s, 1H), 9.83 (m, 1H), 9.46 (s, 1H), 8.23 (s, 1H), 8.15 (d, *J* = 7.2 Hz, 1H), 8.05 (d, *J* = 8 Hz, 1H), 7.47 (s, 1H), 7.26 (t, *J* =8 Hz, 1H), 6.39 (s, 1H), 3.97 (s, 3H), 3.63 (m, 2H), 3.52 (m, 2H), 3.42 (m, 1H), 3.21 (m, 2H), 1.79 (m, 2H), 1.29 (s, 3H), 0.98 (t, *J* = 7.6 Hz, 3H); LCMS calculated for $C_{21}H_{28}N_8O_3$: m/z = 440; found: m/z = 441 $(M+H)^+$.

_N_-(4-(3-(2-morpholinobutyl)ureido)phenyl)-1*H*-indazole-7-carboxamide (BB587)

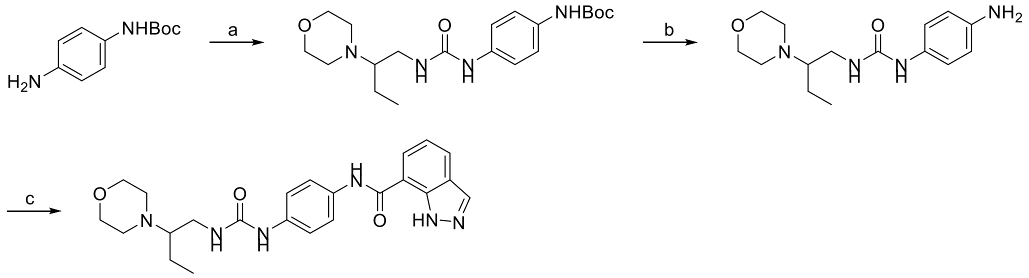

BB0587

Reagents and conditions: (a) 2-morpholinobutan-1-amine, triphosgene, TEA, DCM, 0 °C, 10 min, crude; (b) HCl/MeOH, RT, 1 h, crude; (c) 1*H*-indazole-7-carboxylic acid, DIEA, HATU, DMF, RT, 12 h, 5% yield for 3 steps.

Step a: *tert*-butyl (4-(3-(2-morpholinobutyl)ureido)phenyl)carbamate

To a solution of triphosgene (124 mg, 0.40 mmol, 0.3 eq) in DCM (4 mL) at 0 °C under N_2(g) was added a solution of *tert*-butyl *N*-(4-aminophenyl)carbamate (263 mg, 1.30 mmol, 1.0 eq) in DCM (4 mL). The resulting reaction mixture was stirred at 0 °C for 10 min. Then 2-morpholinobutan-1-amine (0.2 g, 1.26 mmol, 1.0 eq) and TEA (256 mg, 2.53 mmol, 2.0 eq) were added, and the mixture was stirred at 0 °C for 1 h. The reaction mixture was poured into H_2O (10 mL) and extracted with EtOAc/THF (1:1, 3 x 20 mL). The combined organic phase was dried over anhydrous Na_2SO_4 and filtered, and the filtrate was concentrated under reduced pressure to afford *tert*-butyl (4-(3-(2-morpholinobutyl)ureido)phenyl)carbamate (550 mg, crude, 52% purity by LCMS) as a black solid. LCMS calculated for $C_{20}H_{32}N_4O_4$: m/z = 392; found: m/z = 393 $(M+H)^+$. This material was used without further purification.

Step b: 1-(4-aminophenyl)-3-(2-morpholinobutyl)urea

To a solution of HCl/MeOH (4 M, 15 mL) at RT under N_2(g) was added *tert*-butyl *N*-[4-(2

morpholinobutylcarbamoylamino)phenyl]carbamate (200 mg, 0.51 mmol, 1.0 eq). The resulting reaction mixture was stirred at RT for 1 h. The reaction mixture was concentrated under reduced pressure to afford 1-(4-aminophenyl)-3-(2-morpholinobutyl)urea (160 mg, crude, 83% purity by LCMS) as a black solid. LCMS calculated for $C_{15}H_{24}N_4O_2$: m/z = 292; found: m/z = 293 $(M+H)^+$. This material was used without further purification.

Step c: *N*-(4-(3-(2-morpholinobutyl)ureido)phenyl)-1*H*-indazole-7-carboxamide (BB587)

To a solution of 1-(4-aminophenyl)-3-(2-morpholinobutyl)urea (160 mg, 0.54 mmol, 1.0 eq) in DMF (8 mL) at RT under N_2(g) was added DIEA (177 mg, 1.37 mmol, 2.5 eq), HATU (312 mg, 0.82 mmol, 1.5 eq) and 1*H*-indazole-7-carboxylic acid (133 mg, 0.82 mmol, 1.5 eq). The resulting reaction mixture was stirred at RT for 16 h. The reaction mixture was concentrated under reduced pressure to give a residue. The residue was purified by reverse-phase preparative HPLC (column: Waters Xbridge BEH C18 100*30mm*10μm; mobile phase: [water(10mM NH_4HCO_3)-ACN]; B%: 10%-40%, 8 min) to afford *N*-(4-(3-(2-morpholinobutyl)ureido)phenyl)-1*H*-indazole-7-carboxamide (11.8 mg, 5% yield, 96% purity by LCMS) as a white solid. ^{1}H NMR (400 MHz, DMSO-d_6) δ 13.16 (s, br, 1H), 10.27 (s, br, 1H), 8.63 (s, 1H), 8.06 (s, 1H), 8.17 (br s, 1H), 8.05 (d, *J* = 6.8 Hz, 1H), 8.01 (d, *J* = 8 Hz, 1H), 7.67 (d, *J* = 9.2 Hz, 2H), 7.40 (d, *J* = 8.8 Hz, 2H), 7.26 (t, *J* = 7.6 Hz, 1H), 6.03 (m, 1H), 3.63 (m, 4H), 3.25 (m, 1H), 3.24 (m, 1H), 2.55 (m, 3H), 2.35 (m, 2H), 1.55 (m, 1H), 1.25 (m, 1H),1.08 (t, *J* = 7.2 Hz, 3H); LCMS calculated for $C_{23}H_{28}N_6O_3$: m/z = 436; found: m/z = 437 $(M+H)^+$.

***N*-(4-(3-(2-morpholinopropyl)ureido)phenyl)-1*H*-indazole-7-carboxamide (BB588)**

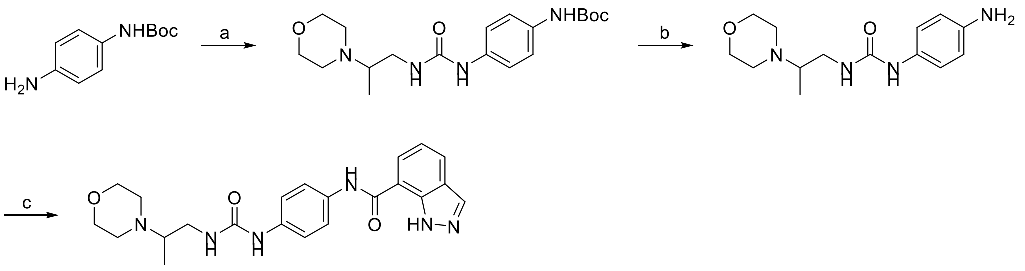

BB0588

Reagents and conditions: (a) 2-morpholinopropan-1-amine, triphosgene, TEA, DCM, 0 °C, 10 min, crude; (b) HCl/MeOH, RT, 1 h, crude; (c) 1*H*-indazole-7-carboxylic acid, DIEA, HATU, DMF, RT, 12 h, 30% yield for 3 steps.

Step a: *tert*-butyl (4-(3-(2-morpholinopropyl)ureido)phenyl)carbamate

To a solution of triphosgene (115 mg, 0.39 mmol, 0.3 eq) in DCM (3.5 mL) at 0 °C under N_2(g) was added a solution of *tert*-butyl *N*-(4-aminophenyl)carbamate (245 mg, 1.18 mmol, 1.0 eq) in DCM (3.5 mL). The resulting reaction mixture was stirred at 0 °C for 10 min. Then 2-morpholinopropan-1-amine (170 mg, 1.18 mmol, 1.0 eq) and TEA (238 mg, 2.36 mmol, 2.0 eq) were added, and the mixture was stirred at 0 °C for 1 h. The reaction mixture was poured into H_2O (15 mL) and extracted with EtOAc/THF (1:1, 3 x 30 mL). The combined organic phase was dried over anhydrous Na_2SO_4 and filtered, and the filtrate was concentrated under reduced pressure to afford *tert*-butyl *N*-[4-(2-morpholinopropylcarbamoylamino)phenyl]carbamate (550 mg, crude, 29% purity by LCMS) as a black solid. LCMS calculated for $C_{19}H_{30}N_4O_4$: m/z = 378; found: m/z = 379 $(M+H)^+$. This material was used without further purification.

Step b: 1-(4-aminophenyl)-3-(2-morpholinopropyl)urea

To a solution of HCl/MeOH (4 M, 20 mL) at RT under N_2(g) was added *tert*-butyl *N*-[4-(2-morpholinopropylcarbamoylamino)phenyl]carbamate (450 mg, 1.19 mmol, 1.0 eq). The resulting reaction mixture was stirred at RT for 1 h. The reaction mixture was concentrated under reduced pressure to afford 1-(4-aminophenyl)-3-(2-morpholinopropyl)urea (330 mg, crude, 86% purity by LCMS) as a black solid.

LCMS calculated for $C_{14}H_{22}N_4O_2$: m/z = 278; found: m/z = 279 $(M+H)^+$. This material was used without further purification.

Step c: *N*-(4-(3-(2-morpholinopropyl)ureido)phenyl)-1*H*-indazole-7-carboxamide (BB588)

To a solution of 1-(4-aminophenyl)-3-(2-morpholinopropyl)urea (330 mg, 1.19 mmol, 1.0 eq) in DMF (5 mL) at RT under N_2(g) was added DIEA (383 mg, 2.96 mmol, 2.5 eq), HATU (676 mg, 1.78 mmol, 1.5 eq) and 1*H*-indazole-7-carboxylic acid (288 mg, 1.78 mmol, 1.5 eq). The resulting reaction mixture was stirred at RT for 12 h. The reaction mixture was concentrated under reduced pressure to give a residue. The residue was purified by reverse-phase preparative HPLC (column: Phenomenex Gemini-NX 80*40mm*3μm; mobile phase: [water(10mM NH_4HCO_3)-ACN]; B%: 10%-40%, 8 min) to afford *N*-[4-(2-morpholinopropylcarbamoylamino)phenyl]-1*H*-indazole-7-carboxamide (157 mg, 30% yield, 95% purity by LCMS) as a black solid. ^{1}H NMR (400 MHz, CD_3OD) δ 8.17 (br s, 1H), 8.04 (m, 2H), 7.68 (d, *J* = 9.2 Hz, 2H), 7.41 (d, *J* = 9.2 Hz, 2H), 7.30 (t, *J* = 7.6 Hz, 1H), 3.73 (m, 4H), 3.33 (m, 1H), 3.32 (m, 1H), 2.65 (m, 3H), 2.54 (m, 2H), 1.06 (d, *J* = 6.8 Hz, 3H); LCMS calculated for $C_{22}H_{26}N_6O_3$: m/z = 422; found: m/z = 423 $(M+H)^+$.

_N_-[5-[(2-methyl-2-morpholino-butyl)carbamoylamino]-2-pyridyl]-1_H_-indazole-7-carboxamide (BB589)

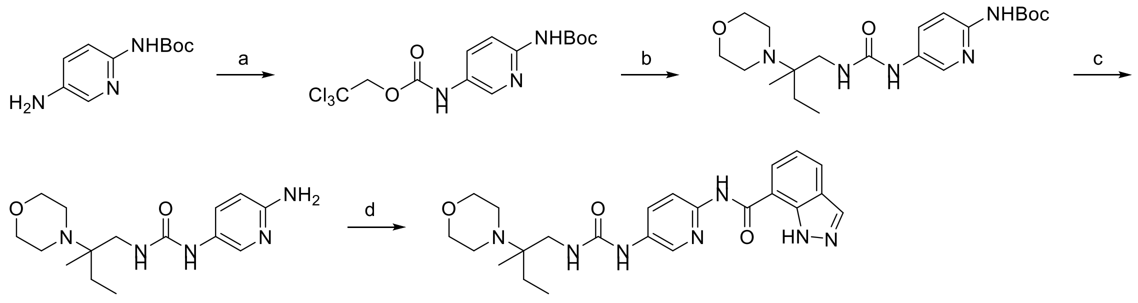

BB0589

Reagents and conditions: (a) 2,2,2-trichloroethyl carbonochloridate, pyridine, THF, 0 °C-RT, 2 h, crude; (b) 2-methyl-2-morpholino-butan-1-amine, DIPEA, DMSO, 70 °C, 12 h, crude; (c) HCl/EtOAc, MeOH, RT, 2 h, crude; (d) 1*H*-indazole-7-carboxylic acid, HATU, TEA, DMF, RT, 6 h, 11.93% yield.

Step a: 2,2,2-trichloroethyl *N*-[6-(*tert*-butoxycarbonylamino)-3-pyridyl]carbamate

To a solution of *tert*-butyl (5-aminopyridin-2-yl)carbamate (1 g, 4.78 mmol, 1 eq) and pyridine (45.36 mg, 573.49 μmol, 46.29 μL, 1.2 eq) in THF (20 mL) was added 2,2,2-trichloroethyl carbonochloridate (1.21 g, 5.73 mmol, 768.98 μL, 1.2 eq) at 0 °C. The suspension was stirred at RT for 2 h, then was poured into water (50 mL) and extracted with ethyl acetate (2 x 30 mL). The combined organic phases were washed with brine (50 mL), dried over anhydrous Na_2SO_4, filtered, and concentrated in vacuum to afford 2,2,2-trichloroethyl *N*-[6-(*tert*-butoxycarbonylamino)-3-pyridyl]carbamate (2 g, crude) as a white solid. ^{1}H NMR (400 MHz, DMSO-d_6) δ 10.20 (br s, 1H), 9.67 (s, 1H), 8.34 (br s, 1H), 7.84 (br d, *J* = 8.4 Hz, 1H), 7.73 (d, *J* = 8.8 Hz, 1H), 4.93 (s, 2H), 1.46 (s, 9H); LCMS calculated for $C_{13}H_{16}Cl_3N_3O_4$: m/z = 384; found: m/z = 385 $(M+H)^+$. The crude product was used in the next step without further purification.

Step b: *tert*-butyl *N*-[5-[(2-methyl-2-morpholino-butyl)carbamoylamino]-2-pyridyl]carbamate

To a solution of 2,2,2-trichloroethyl *N*-[6-(*tert*-butoxycarbonylamino)-3-pyridyl]carbamate (1 g, 2.60 mmol, 1 eq) in DMSO (10 mL) was added DIPEA (436.81 mg, 3.38 mmol, 588.69 μL, 1.3 eq) and 2-methyl-2-morpholino-butan-1-amine (582.22 mg, 3.38 mmol, 1.3 eq). The solution was stirred at 70 °C for 12 h, then was poured into water (30 mL) and extracted with ethyl acetate (3 x 20 mL). The combined organic phases were washed with brine (20 mL), dried over anhydrous Na_2SO_4, filtered, and concentrated in vacuum. The residue was purified by flash silica gel chromatography (ISCO®; 20 g SepaFlash® silica flash column, 0-

80% ethyl acetate/petroleum ether gradient at 60 mL/min) to afford *tert*-butyl *N*-[5-[(2-methyl-2-morpholino-butyl)carbamoylamino]-2-pyridyl]carbamate (440 mg) as a yellowish solid. ^{1}H NMR (400 MHz, DMSO-d_6) δ 9.54 (m, 1H), 8.74 (s, 1H), 8.27 (m, 1H), 7.78 (m, 1H), 7.69 (m, 1H), 5.97 (m, 1H), 3.58 (br t, *J* = 4.4 Hz, 4H), 3.42 (m, 3H), 3.32 (br s, 2H), 3.09 (m, 1H), 1.46 (m, 12H), 0.88 (m, 2H), 0.81 (t, *J* = 7.6 Hz, 2H). The crude product was used in the next step without further purification.

Step c: 1-(6-amino-3-pyridyl)-3-(2-methyl-2-morpholino-butyl)urea

To a solution of *tert*-butyl *N*-[5-[(2-methyl-2-morpholino-butyl)carbamoylamino]-2-pyridyl] carbamate (390 mg, 957.04 µmol, 1 eq) in MeOH (5 mL) was added HCl/EtOAc (10 mL). The solution was stirred at RT for 2 h, then was concentrated under reduced pressure to remove the solvent. 1-(6-amino-3-pyridyl)-3-(2-methyl-2-morpholino-butyl)urea (crude, 350 mg, HCl salt) was obtained as a brown solid. ^{1}H NMR (400 MHz, DMSO-d_6) δ 8.20 (s, 1H), 7.89 (dd, *J* = 9.6, 2.4 Hz, 1H), 7.01 (d, *J* = 7.2 Hz, 1H), 4.09 (m, 2H), 3.89 (m, 2H), 3.64 (m, 3H), 3.35 (s, 2H), 3.27 (m, 1H), 1.85 (m, 2H), 1.37 (s, 3H), 1.07 (t, *J* = 7.6 Hz, 3H). The crude product was used in the next step without further purification.

Step d: *N*-[5-[(2-methyl-2-morpholino-butyl)carbamoylamino]-2-pyridyl]-1*H*-indazole-7-carboxamide (BB589)

To a solution of 1*H*-indazole-7-carboxylic acid (101.86 mg, 628.18 µmol, 1.2 eq) in DMF (2 mL) was added HATU (238.85 mg, 628.18 µmol, 1.2 eq) and TEA (211.88 mg, 2.09 mmol, 291.45 µL, 4 eq). The solution was stirred at 20 °C for 10 min, then 1-(6-amino-3-pyridyl)-3-(2-methyl-2-morpholino-butyl)urea (0.18 g, 523.48 µmol, 1 eq, HCl salt) was added. The solution was stirred at RT for 6 h, then was poured into water (30 mL) and extracted with ethyl acetate (2 x 20 mL). The combined organic phases were washed with brine (20 mL), dried over anhydrous Na_2SO_4, filtered, and concentrated in vacuum. The residue was purified by prep-HPLC (neutral condition: column: Waters Xbridge BEH C18 100*25mm*5µm; mobile phase: [water(10 mM NH_4HCO_3)-ACN]; B%: 20%-50%, 10 min) to give the impure product (60 mg, purity 83% by HPLC) as a light yellow solid. The product was triturated with DMF (5 mL) at 20 °C for 10 min and filtered, and the filter cake was washed with H_2O (3 mL). After lyophilization, *N*-[5-[(2-methyl-2-morpholino-butyl)carbamoylamino]-2-pyridyl]-1*H*-indazole-7-carboxamide (30.2 mg, 62.44 µmol, 11.93% yield, 93.36% purity) was obtained as a white solid. ^{1}H NMR (400 MHz, CD_3OD) δ 8.46 (s, 1H), 8.37 (m, 1H), 8.19 (m, 3H), 7.95 (m, 1H), 7.31 (t, *J* = 7.6 Hz, 1H), 4.13 (m, 2H), 3.85 (m, 2H), 3.68 (m, 2H), 3.58 (m, 2H), 1.84 (m, 2H), 1.38 (s, 3H), 1.08 (br t, *J* = 7.2 Hz, 3H); ^{1}H NMR (400 MHz, DMSO-d_6) δ 13.20 (br s, 1H), 10.82 (s, 1H), 9.52 (m, 1H), 9.31 (br s, 1H), 8.46 (br s, 1H), 8.20 (m, 2H), 8.02 (br d, *J* = 8 Hz, 1H), 7.92 (m, 1H), 7.19 (m, 2H), 3.95 (m, 4H), 3.56 (m, 3H), 3.42 (m, 1H), 3.20 (m, 2H), 1.79 (m, 2H), 1.28 (s, 3H), 0.98 (br t, *J* = 7.2 Hz, 3H); LCMS calculated for $C_{23}H_{29}N_7O_3$: m/z = 451; found: m/z = 452 $(M+H)^+$.
